# Validation of Immunotherapy Response Score as Predictive of Pan-solid Tumor Anti-PD-1/PD-L1 Benefit

**DOI:** 10.1158/2767-9764.CRC-23-0036

**Published:** 2023-07-25

**Authors:** Benjamin J. Bulen, Nickolay A. Khazanov, Daniel H. Hovelson, Laura E. Lamb, Marc Matrana, Mark E. Burkard, Eddy Shih-Hsin Yang, William J. Edenfield, Elizabeth Claire Dees, Adedayo A. Onitilo, Gary L. Buchschacher, Alan M. Miller, Benjamin M. Parsons, Timothy R. Wassenaar, Jennifer M. Suga, Robert D. Siegel, William Irvin, Suresh Nair, Jennifer N. Slim, Jamal Misleh, Jamil Khatri, Gregory A. Masters, Sachdev Thomas, Malek M. Safa, Daniel M. Anderson, Jonathan Mowers, Anna C. Dusenbery, Stephanie Drewery, Komal Plouffe, Travis Reeder, Hana Vakil, Lynnae Patrias, Amanda Falzetta, Ryan Hamilton, Kat Kwiatkowski, D. Bryan Johnson, Daniel R. Rhodes, Scott A. Tomlins

**Affiliations:** 1 Strata Oncology, Ann Arbor, Michigan.; 2 Ochsner Cancer Institute, New Orleans, Louisiana.; 3 University of Wisconsin Carbone Cancer Center, Madison, Wisconsin.; 4 O'Neal Comprehensive Cancer Center, University of Alabama at Birmingham School of Medicine, Birmingham, Alabama.; 5 Prisma Health Greenville Memorial Hospital, Greenville, Carolina.; 6 UNC Lineberger Comprehensive Cancer Center, Chapel Hill, North Carolina.; 7 Cancer Care and Research Center, Marshfield Clinic Research Institute, Marshfield, Wisconsin.; 8 Kaiser Permanente Southern California, Los Angeles, California.; 9 Translational Drug Development, Scottsdale, Arizona.; 10 Gundersen Health System, La Crosse, Wisconsin.; 11 UW Health Cancer Center at ProHealth Care, Waukesha, Wisconsin.; 12 Kaiser Permanente Northern California, Vallejo, California.; 13 Bon Secours St. Francis Cancer Center, Greenville, Carolina.; 14 Bon Secours Cancer Institute, Midlothian, Virginia.; 15 Lehigh Valley Topper Cancer Institute, Allentown, Pennsylvania.; 16 MultiCare Regional Cancer Center – Tacoma, Washington.; 17 The US Oncology Network, Newark, Delaware.; 18 ChristianaCare Oncology Hematology, Newark, Delaware.; 19 Medical Oncology Hematology Consultants, Helen F Graham Cancer Center and Research Institute, Newark, Delaware.; 20 Kaiser Permanente Northern California, Oakland, California.; 21 Kettering Health, Kettering, Ohio.; 22 Metro-Minnesota Community Oncology Research Consortium, St. Louis Park, Minnesota.

## Abstract

Immunotherapy response score (IRS) integrates tumor mutation burden (TMB) and quantitative expression biomarkers to predict anti-PD-1/PD-L1 [PD-(L)1] monotherapy benefit. Here, we evaluated IRS in additional cohorts. Patients from an observational trial (NCT03061305) treated with anti-PD-(L)1 monotherapy were included and assigned to IRS-High (-H) versus -Low (-L) groups. Associations with real-world progression-free survival (rwPFS) and overall survival (OS) were determined by Cox proportional hazards (CPH) modeling. Those with available PD-L1 IHC treated with anti-PD-(L)1 with or without chemotherapy were separately assessed. Patients treated with PD-(L)1 and/or chemotherapy (five relevant tumor types) were assigned to three IRS groups [IRS-L divided into IRS-Ultra-Low (-UL) and Intermediate-Low (-IL), and similarly assessed]. In the 352 patient anti-PD-(L)1 monotherapy validation cohort (31 tumor types), IRS-H versus IRS-L patients had significantly longer rwPFS and OS. IRS significantly improved CPH associations with rwPFS and OS beyond microsatellite instability (MSI)/TMB alone. In a 189 patient (10 tumor types) PD-L1 IHC comparison cohort, IRS, but not PD-L1 IHC nor TMB, was significantly associated with anti-PD-L1 rwPFS. In a 1,103-patient cohort (from five relevant tumor types), rwPFS did not significantly differ in IRS-UL patients treated with chemotherapy versus chemotherapy plus anti-PD-(L)1, nor in IRS-H patients treated with anti-PD-(L)1 versus anti-PD-(L)1 + chemotherapy. IRS associations were consistent across subgroups, including both Europeans and non-Europeans. These results confirm the utility of IRS utility for predicting pan-solid tumor PD-(L)1 monotherapy benefit beyond available biomarkers and demonstrate utility for informing on anti-PD-(L)1 and/or chemotherapy treatment.

**Significance:**

This study confirms the utility of the integrative IRS biomarker for predicting anti-PD-L1/PD-1 benefit. IRS significantly improved upon currently available biomarkers, including PD-L1 IHC, TMB, and MSI status. Additional utility for informing on chemotherapy, anti-PD-L1/PD-1, and anti-PD-L1/PD-1 plus chemotherapy treatments decisions is shown.

## Introduction

The durable clinical benefit of monoclonal antibodies against PDCD1 (PD-1) and CD274 (PD-L1) [together PD-(L)1] in selected patients has revolutionized the care of patients with advanced cancer, with approvals in multiple tumor types and pan tumor indications [microsatellite instability high (MSI-H)/mismatch repair deficient] and tumor mutation burden high [TMB-H; ≥10 mutations/megabase (Muts/Mb)] (1–3). While MSI and TMB are important pan tumor biomarkers, they fail to identify most patients who benefit from anti-PD-(L)1 therapy. In the KEYNOTE-158 study of 10 tumor types leading to pan-solid tumor approval of pembrolizumab (anti-PD-1) monotherapy in second-line or greater line TMB-H patients, while a higher objective response rate was observed in those TMB-H (29%) versus TMB-Low (-L; 6%), numerically more objective responses were observed in those TMB-L (43/688) versus TMB-H (30/102; ref. 4), consistent with similar studies indicating that TMB-H alone misses most anti-PD(L)1 therapy-responsive patients (5). In addition, a recent report by Nassar and colleagues highlighted the potential limitations of TMB from tumor-only comprehensive genomic profiling (CGP) for predicting checkpoint inhibitor benefit, due to overestimation of TMB-H (from misclassification of germline variants as somatic mutations) status (6, 7), particularly in patients with non-European ancestry (8). Similarly, although PD-L1 IHC is another important anti-PD-(L)1 therapy biomarker, particularly in first-line treatment decisions, in practice it does not represent a pan tumor biomarker, but rather a series of tumor type–specific biomarkers with various antibodies, staining platforms, PD-L1–expressing cells included in scoring algorithms, and cutoffs (9–19).

We recently reported the development and validation of an integrated immunotherapy response score (IRS) algorithm using deidentified clinical and molecular data maintained in the Strata Clinical Molecular Database (SCMD) from patients in the Strata Trial (NCT03061305), an observational clinical trial evaluating the impact of tumor molecular profiling for patients with advanced solid tumors (20). IRS combines TMB with four target gene expression measurements (*PD-L1*, *PD-1*, *TOP2A,* and *ADAM12)* from simultaneous, analytically valid, CGP plus quantitative transcriptional profiling (qTP) from formalin-fixed paraffin-embedded (FFPE) tumor specimens. IRS status [-High, more likely to benefit from anti-PD-(L)1 therapy; (-H) vs. -Low (-L)] was validated to predict PD-(L)1 monotherapy treatment benefit by both time to next therapy, a validated real-world progression-free survival (rwPFS) endpoint, and overall survival (OS; ref. 20).

Importantly, early line anti-PD-(L)1 development has largely moved to a combination strategy based on proposed synergy between anti-PD-(L)1 and other therapy classes, including chemotherapy (21, 22). However, analyzing the clinical activity of individual components of 13 phase III anti-PD-(L)1 combination trials (and other relevant trials of individual components) across tumor types, Palmer and colleagues found no evidence of synergy between individual agents, instead concluding that the individual components benefit distinct patient populations, with this strategy offering patients multiple chances of response, highlighting the need for better predictors of anti-PD-(L)1 benefit to guide treatment decisions beyond anti-PD-L1 monotherapy (23). Notably, 35.0% of the IRS discovery cohort was treated with pembrolizumab combination, and in a propensity-matched analysis of patients with first-line non–small cell lung cancer (NSCLC), IRS-L patients had significantly longer rwPFS on pembrolizumab + chemotherapy versus pembrolizumab, while rwPFS did not significantly differ in IRS-H patients treated with pembrolizumab + chemotherapy versus pembrolizumab. These results suggest IRS may have additional utility for informing on anti-PD-(L)1 and/or chemotherapy benefit prediction where relevant.

Hence, herein we evaluated IRS performance for predicting PD-(L)1 monotherapy (including pembrolizumab specifically) benefit in a second, independent validation cohort of Strata Trial patients in the SCMD. We also evaluated the ability of IRS to add to both currently available CGP biomarkers (TMB and MSI) and PD-L1 IHC. In addition, we assessed the robustness of IRS (and the included TMB component) across self-reported racial groups in the overall SCMD and in non-European patients treated with PD-(L)1 monotherapy. Finally, we sought to determine the utility of IRS status for anti-PD-L1 and/or chemotherapy benefit prediction by assessing the previously described three-group IRS status [where IRS-L is divided into IRS-Intermediate-Low (-IL) and Ultra-low (-UL) (20)] across a cohort of patients treated with anti-PD-(L)1 monotherapy, chemotherapy, or anti-PD-(L)1 + chemotherapy in relevant tumor types.

## Materials and Methods

### Cohort

The Strata Trial (NCT03061305) and the SCMD have been described previously (20). Briefly, at enrolling health systems, all adult (ages ≥18 years old) patients with unresectable or metastatic solid tumors and available FFPE tumor tissue are eligible. The Strata Trial has been reviewed and approved by Advarra Institutional Review Board (IRB; IRB Pro00019183) prior to study start. All patients provided written informed consent for Strata Trial participation, except at institutions where a waiver of informed consent was granted by the central and/or local IRB and applied because of minimal risk of using surplus tissue specimens. This study was performed in accordance with the Declaration of Helsinki and we have complied with all relevant ethical regulations. Additional details are provided in the Supplementary Methods S1.

For the anti-PD-(L)1 monotherapy validation cohort, inclusion/exclusion criteria were the same as the original IRS discovery/validation cohorts (20): (i) reportable TMB measurements from StrataNGS testing (including meeting the overall 20% tumor content requirement), (ii) reportable immune gene expression quantification from an investigative multiplex PCR-based transcriptomic profiling (qTP) test, (iii) treatment with a systemic pembrolizumab or other PD-(L)1 monotherapy line of therapy, (iv) the tested tissue specimen was collected prior to the PD-(L)1 monotherapy start date, and (v) the subject had had no prior anti-PD-(L)1 or CTLA4 blockade therapy prior to the PD-(L)1 monotherapy line start date. All patients included in the original discovery or validation cohorts were specifically excluded. rwPFS for each therapy line was defined as the time from starting the line to the time of stopping that line and starting a new therapy line or death (patients without one of these two events were censored at date of last follow-up) as described previously (20). Both rwPFS and OS were used for studying treatment outcome as in the original IRS validation cohort. Patients in the SCMD tested by a version of StrataNGS assessing TMB with parallel gene expression testing data completed from January 25, 2017 to April 25, 2023 were eligible for analysis with a data cutoff of April 25, 2023.

For the self-reported race validation cohort, criteria (i)–(v) as above were the same, patients who were MSI-H were additionally excluded [as TMB inflation could confound both the TMB component (from the CGP component of Strata Select; ref. 24) and IRS status, but would have no impact on MSI status], and only patients included in the original discovery cohort were excluded (to maximize the number of non-European patients for analysis). Given the limited number of non-European patients, only rwPFS was assessed.

For the PD-L1 IHC cohort, criteria (i)–(v) as above were the same, and patients in the original discovery cohort were included [to maximize the number of patients with available clinical PD-L1 IHC in accompanying pathology reports (Supplementary Methods S1)]. Given the cohort size, only rwPFS wasbrk assessed.

For the anti-PD-(L)1 and/or chemotherapy validation cohort, inclusion/criteria (i)–(v) were as above, except for (iii) eligible treatment lines were PD-(L)1 monotherapy, chemotherapy, or PD-(L)1 + chemotherapy; for (iv) the specimen was collected prior to the included line(s) start date; and (v) the subject had no prior anti-PD-(L)1 or CTLA4 blockade therapy prior to the included line(s) start date. Only tumor types where (i) first-line chemotherapy with or without anti-PD-(L)1 is National Comprehensive Cancer Network (NCCN) recommended and (ii) later-line anti-PD-(L)1 with or without chemotherapy is also recommended were considered. Combination lines with ramucirumab, bevacizumab or cetuximab were included. For NSCLC, single-agent chemotherapy lines were excluded; for esophagogastric cancer (EGC), all lines including anti-HER2 targeted agents were excluded. All PD-(L)1 ± chemotherapy lines used in IRS discovery were specifically excluded. For inclusion of a tumor type, we targeted ≥80 total lines [at least 40 chemotherapy lines, 20 chemotherapy + anti-PD-(L)1 lines, and 20 PD-(L)1 monotherapy lines] resulting in five final included tumor types that met these criteria except as noted: NSCLC, head and neck cancer [H&N; only *n* = 6 anti-PD-(L)1 + chemotherapy lines], EGC, small cell lung cancer (SCLC), and triple-negative breast cancer (TNBC) [only *n* = 13 anti-PD-(L)1 lines]. Only rwPFS was assessed given the desire to directly compare treatment line benefit without the confounder of benefit from later line [e.g., anti-PD-(L)1 vs. earlier chemotherapy] treatment.

### Biomarker Data

Multiplex PCR-based CGP and in parallel immune gene expression by an analytically and clinically validated multiplex PCR-based investigational/supplementary qTP test (now available as Strata Select) was performed on coisolated DNA and RNA from FFPE solid tumor tissue (Strata Oncology) as described previously (20, 24). Additional analytic validity analyses of the gene expression component of IRS were performed as described in the Supplementary Methods S1. Individual patient IRS was derived from the Cox model as in the original discovery and validation cohort: IRS = 0.273758 * TMB + 0.112641 * *PD-1* + 0.061904 * *PD-L1* − 0.077011 * *TOP2A* − 0.057991 * *ADAM12.* All patients were assigned one of two IRS groups to compare patient outcomes using the previously validated threshold [i.e., Low (L) < 0.873569 and High (H) ≥0.873569; more likely to benefit from anti-PD-(L)1 therapy (20)]. For the PD-(L)1 and/or chemotherapy validation cohort, IRS-L was further divided into IRS-UL (< 0.41) and IRS-IL (0.41 to < 0.873569), as described previously (20).

### Statistical Analysis

The power analysis to identify the appropriate size of a second monotherapy validation cohort was the same as in the previous IRS validation (20). Briefly, in the original discovery cohort, 46% patients were IRS-H, and we observed an adjusted HR (aHR) for IRS-H versus IRS-L rwPFS of 0.49 (47% event rate); therefore, assuming an IRS-H to IRS-L ratio of 1:1 and a 50% event rate, a validation cohort of 180 patients would have 90% power to detect a similar (0.5) HR at an alpha of 0.05. We therefore identified all (*n* = 352) patients in the SCMD meeting the above-described inclusion/exclusion criteria; notably, the pembrolizumab-treated subset was of sufficient size (*n* = 288) to also meet the above criteria. The IRS algorithm (and -H vs. -L threshold) was then applied to these subjects in the anti-PD-(L)1 monotherapy validation cohort. No power calculation was performed for the self-reported race validation cohort, PD-L1 IHC cohort, or anti-PD-(L)1 and/or chemotherapy validation cohort.

For all cohorts, unadjusted rwPFS and OS across groups and treatments were visualized using the Kaplan–Meier method. Adjusted rwPFS and OS analyses were performed to compare group outcomes (by aHRs and two-sided *P* values) using Cox proportional hazards models unless otherwise specified. Covariate adjustments shared between all models applied to the anti-PD-(L)1 monotherapy validation cohort (unless otherwise noted) include age (in years at the time of tested sample collection), sex assigned at birth (sex as a biological variable is included as part of sample level quality control for StrataNGS testing), tumor type (all tumor types with >15 samples in the monotherapy validation cohort were considered separately; remaining tumor types were grouped into a single category), line of systemic therapy (continuous), and pembrolizumab versus other PD-(L)1 therapy, and IRS-H versus -L status,. Sensitivity analyses were performed using the same base Cox model as appropriate for assessment of individual variables. For the predictive analysis using the case–control internal comparator cohort subset, we compared rwPFS on the immediately preceding systemic therapy versus subsequent PD-(L)1 monotherapy to examine the interaction between PD-(L)1 versus prior therapy rwPFS within the same patient and IRS status (IRS-H vs. IRS-L), as well as compared the rate of rwPFS2 [PD-(L)1]/rwPFS1 (prior therapy) ≥1.3 (25–27) in IRS-H versus IRS-L patients. Additional details and modifications to the included covariates for the remaining cohorts are provided in the Supplementary Methods S1.

Self-reported race was collected at Strata Trial enrollment using provided categories and is reported from the overall population of 24,463 patients in the SCMD where IRS could be generated (regardless of treatment data availability) at the time of IRS development (20). Patients self-reporting as American Indian or Alaskan Native, or Native Hawaiian or Other Pacific Islander, or Other were combined into a single group (“Other”), and patients self-reporting as Other, Asian, or Black or African American were analyzed both individually and collectively (“non-European”) versus White or Caucasian (“European”) as in ref. 8. The frequency of TMB-H and IRS-H status across the SCMD, as well as for selected primary tumor types (as in ref. 8) was determined by these self-reported racial groups. Additional details are provided in the Supplementary Methods S1.

Throughout this study, TMB-H was defined as ≥10 Muts/Mb by StrataNGS, given the previous validation of TMB by StrataNGS and concordance with TMB estimates from FoundationOne tissue testing (24). All statistical analyses were performed in R (v. 4), SAS (v. 9.4), and MedCalc (v20). For all analyses, two-sided *P* values <0.05 were considered statistically significant. The REMARK checklist for the study is provided in Supplementary Data S1.

### Data Availability

Because of applicable data sharing agreements and/or patient-informed consent forms with Strata Trial health care systems and participants, the authors are restricted from making raw patient-level genomic sequencing data publicly available or deposited. Interested parties may contact the authors at BD@strataoncology.com to request access for research purposes, and such requests will be handled on a case-by-case basis. All clinical and treatment data for the validation cohort described herein is available in Supplementary Data S2; similar data from the previous validation cohort used herein is available in ref. 20.

The IRS model algorithm is available through GitHub: https://github.com/StrataOncology/immune-response-score. All other data are available from the corresponding author on reasonable request.

## Results

### Validation of IRS for Predicting Anti-PD-(L)1 Monotherapy Benefit

**FIGURE 1:**
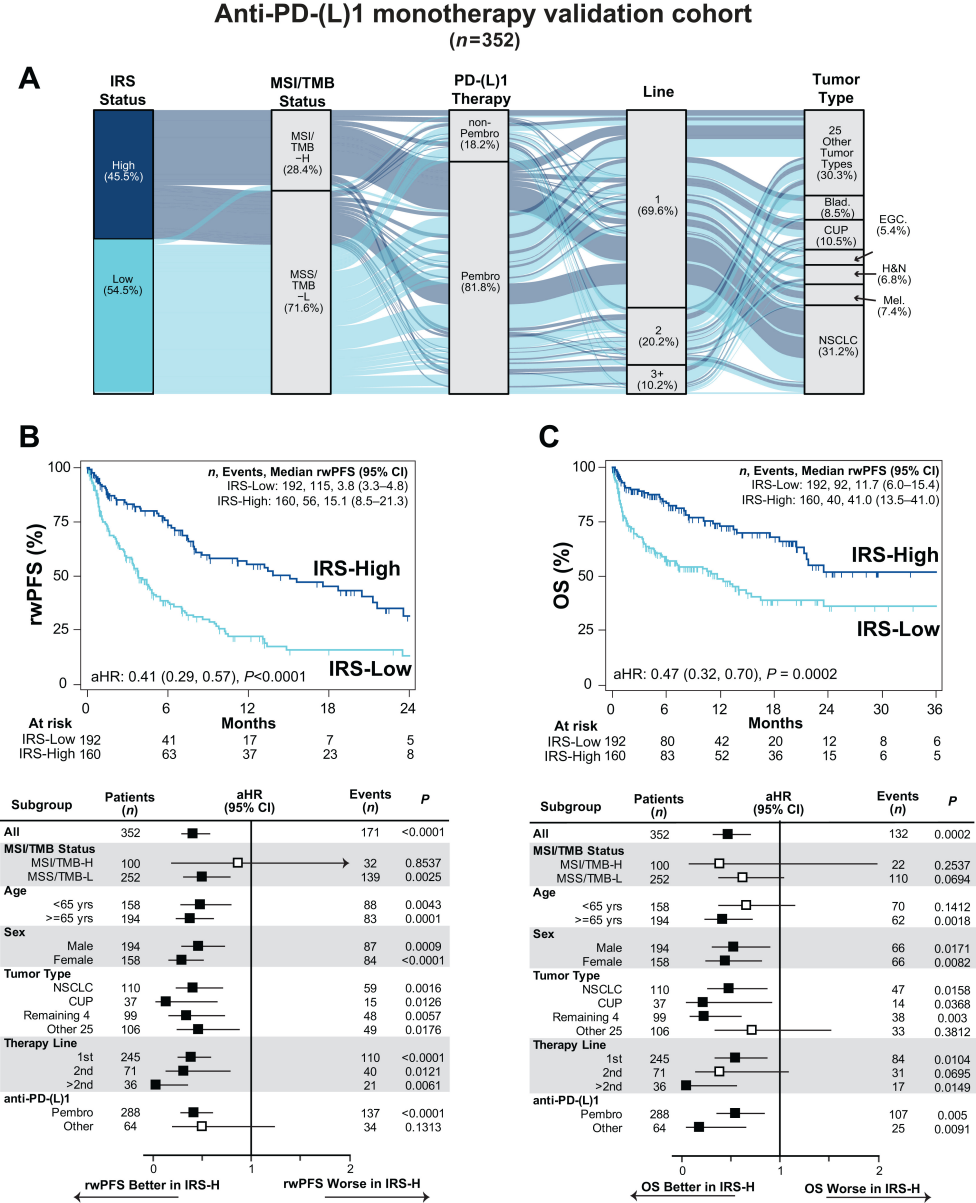
Validation of IRS to stratify anti-PD-(L)1 monotherapy benefit in patients with advanced solid tumors. **A,** Clinical characteristics of the anti-PD-(L)1 monotherapy validation cohort are shown in an alluvial diagram. All patients with available clinical molecular profiling data necessary for IRS (TMB and normalized expression of *PD-1*, *PD-L1*, *ADAM12,* and *TOP2A* from in-parallel qTP) from FFPE tumor tissue enrolled in the Strata Trial (NCT03061305) and treated with systemic anti-PD-(L)1 monotherapy were considered. Patients in previous IRS discovery or validation were excluded. The locked IRS model and thresholds were used to assign IRS-L (light blue) or IRS-H (increased benefit; dark blue) status. For the 352 eligible patients, IRS status, MSI/TMB status (MSI-H or TMB-H as MSI/TMB-H), type of anti-PD-(L)1 therapy [pembrolizumab (pembro) vs. other anti-PD-(L)1], systemic line of anti-PD-(L)1 therapy, and tumor type [all tumor types with >15 samples considered individually: NSCLC, cancer of unknown primary (CUP), bladder cancer (Blad.), melanoma (Mel.), head and neck cancer (H&N), and EGC; remaining 25 other tumor types considered together] are shown. Stratum are colored by IRS status. IRS stratifies anti-PD-(L)1 monotherapy clinical benefit by rwPFS (by time to next therapy; B) and OS (C). **B,** Anti-PD-(L)1 monotherapy rwPFS stratified by IRS group is shown by unadjusted Kaplan–Meier analysis, with the aHR [adjusted for age, sex assigned at birth, line of therapy, tumor type and anti-PD-(L)1 therapy type], 95% CI and *P* value for IRS status (IRS-H vs. IRS-L) shown. The number (*n*) of patients, events, and median rwPFS (with 95% CI) for each group are shown. Forest plot analyses of rwPFS by IRS status in key subgroups are shown below (Remaining 4 = Blad., Mel., H&N, and EGC). Significant associations are shown by filled in aHR estimates. **C,** As in B, except assessing OS.

We previously developed and validated the integrative IRS using deidentified clinical and molecular data from patients in the Strata Trial (NCT03061305) maintained the SCMD (20). Additional analytic validity analyses demonstrating Pearson correlation coefficient (*r*) of 0.946–0.969 and 1,757–47,803-fold linearity (vs. qRT-PCR) for the individual normalized gene expression components of IRS, including *PD-L1* expression, are shown in Supplementary Fig. S1. Herein, we first sought to validate IRS performance in a second, independent validation cohort of NCT03061305 patients treated with anti-PD-L1 monotherapy (including a sufficiently powered pembrolizumab cohort). With a data cutoff of April 25, 2023, the SCMD contained clinical, molecular, and at least one systemic antineoplastic treatment record from a total of 11,212 unique patients with advanced solid tumors (from 43 tumor types) from 31 U.S. health care systems who had routine FFPE tumor tissue molecularly profiled by the StrataNGS CGP test and an in parallel qTP test (20). Of these, 352 (3.1%) met all inclusion/exclusion criteria for this anti-PD-(L)1 monotherapy validation cohort, including not being in the original discovery or validation cohorts (Supplementary Fig. S2). As shown in Fig. 1A, this cohort was comprised of 352 patients with 31 tumor types, with NSCLC being the most frequent (31.2%); 100 (28.4%) patients were TMB-H or MSI-H by StrataNGS testing (tumor types and demographics are provided in Supplementary Table S1). Median total follow-up in these 352 patients was 9.1 months, with 171 (48.6%) real-world progression events and 132 deaths (37.5%); in the 288 treated with pembrolizumab, median follow-up was 9.25 months, with 47.6% real-world progression events and 37.2% deaths.

Therefore, the 352 patients were assigned IRS status using the previously validated IRS-H versus -L threshold [*n* = 160 (45.5%) -H and *n* = 192 (54.5%) -L, Fig. 1A; 45.8% IRS-H in those treated with pembrolizumab], and we compared group outcomes by Kaplan–Meier analysis (unadjusted) and Cox proportional hazards modeling (Supplementary Table S2). Proportional hazards assumptions were checked (for this and all subsequent analyses) using Schoenfeld residuals, with unstratified results presented, as stratification to preserve proportional hazards (where the assumption was not met) produced similar covariate effect sizes. As shown in Fig. 1B and C, IRS-H patients had significantly longer PD-(L)1 rwPFS [median rwPFS 15.1 (95% confidence interval, CI, 8.5–21.3) vs. 3.8 (95% CI, 3.3–4.8) months, aHR 0.41 (95% CI, 0.29–0.57), *P* < 0.0001; Fig. 1B)] and OS [IRS-H vs. IRS-L median OS 41.0 (95% CI, 13.5–41.0) vs. 11.7 (95% 6.0–15.4) months, aHR 0.47 (95% CI, 0.32–0.70), *P* = 0.0002; Fig. 1C]. We also confirmed IRS-H patients also showed significant rwPFS and OS benefit compared with IRS-L patients when using restricted mean survival time analysis (Supplementary Table S3), which does not require the proportional hazards assumption to be met.

Importantly, subgroup analyses showed consistent results across key patient subsets, most notably across tumor types, those not MSI/TMB-H [microsatellite stable (MSS)/TMB-L], as well as those treated with pembrolizumab (Fig. 1B and C). Notably, however, IRS status was not significantly associated with rwPFS (nor OS) in the MSI/TMB-H subset of patients (Fig. 1B and C), consistent with their already high likelihood of substantial anti-PD-(L)1 monotherapy benefit and the limited number of MSI/TMB-H but IRS-L patients (Fig. 1A; Supplementary Fig. S3A and S3B).

To demonstrate the added value of IRS status beyond TMB/IRS-H status alone, we first assessed the MSS/TMB-L subset of patients, where those IRS-H had significantly longer rwPFS versus those IRS-L [median rwPFS 11.2 (95% CI, 6.2–18.7) vs. 3.8 (95% CI 2.8–4.8) months, aHR 0.50 (95% CI, 0.31–0.78), *P* = 0.0025; Supplementary Fig. S3C] and numerically longer OS median OS 21.6 (95% CI, 11.2–41.0) vs. 11.7 (95% CI, 5.5–15.4) months, aHR 0.62 (95% CI, 0.37–1.04), *P* = 0.069; Supplementary Fig. S3D). In addition, adding IRS status to MSI/TMB-H status (MSI/TMB-H, MSS/TMB-L/IRS-H, and MSS/TMB-L/IRS-L), resulted in MSS/TMB-L/IRS-H versus MSS/TMB-L/IRS-L being independently associated with rwPFS and OS [aHR 0.47 (95% CI, 0.30–0.74), *P* = 0.001 and aHR 0.59 (95% CI, 0.36–0.98), *P* = 0.04 respectively; Supplementary Fig. S3E and S3F] and improved the significance of the Cox model association with rwPFS and OS compared with MSI/TMB-H status alone [likelihood ratio test (LRT) *P* = 0.0006 and *P* = 0.03, respectively; Supplementary Fig. S3E and S3F]. Taken together, these results confirm that IRS predicts anti-PD-(L)1 monotherapy benefit (including pembrolizumab), independent of currently available pan-solid tumor CGP biomarkers.

### Confirmation of the Predictive Nature of IRS

In our previous study, we established the IRS algorithm as predictive (vs. prognostic) in through both showing that IRS was not predictive on non-immunotherapy first-line systemic rwPFS, as well as through a case cross-over study in the 146 discovery cohort patients who had received a previous line of systemic therapy prior to pembrolizumab monotherapy. Hence, we sought to confirm these results in this anti-PD-(L)1 monotherapy validation cohort through a case cross-over analysis in the 107 of 352 (30.4%) patients with 24 tumor types who were treated with systemic therapy prior to PD-(L)1 monotherapy (Supplementary Fig. S2; Supplementary Table S4).

As shown in Supplementary Fig. S4, while PD-(L)1 compared with the immediately preceding therapy line rwPFS did not significantly differ in IRS-L patients [*n* = 73, IRS-L PD-(L)1 vs. immediately preceding therapy median rwPFS 3.7 (95% CI, 2.2–4.5) vs. 5.2 (95% CI, 3.5–5.6) months, log-rank *P* = 0.39; Supplementary Fig. S4A], PD-(L)1 rwPFS was significantly longer than the immediately preceding therapy line in IRS-H patients [*n* = 34, IRS-H PD-(L)1 vs. immediately preceding therapy median rwPFS 20.5 (95% CI, 15.6–20.5) vs. 4.9 (95% CI, 3.2–5.6) months, log-rank *P <* 0.0001; Supplementary Fig. S4A]. The test for interaction between PD-(L)1 versus immediately preceding treatment line and IRS status (IRS-H vs. IRS-L) was significant (LRT for interaction *P <* 0.0001). Notably, as shown in Supplementary Fig. S4B, results were similar in an exploratory analysis when limiting the cohort to the *n* = 86/352 (24.4%) MSS/TMB-L patients [*n* = 34 IRS-L PD-(L)1 vs. immediately preceding therapy median rwPFS 3.7 (95% CI, 2.2–4.5) vs. 5.2 (95% CI, 3.9–5.6) months, log-rank *P* = 0.36; *n* = 14 IRS-H PD-(L)1 vs. immediately preceding therapy median rwPFS 18.7 (95% CI, 0.9–18.7) vs. 4.9 (95% CI, 2.7–7.3) months, log-rank *P* = 0.15; LRT for interaction *P* = 0.009]. Likewise, in an exploratory analysis based on type of previous therapy [chemotherapy alone, chemotherapy combined with another class of therapy, and non-chemotherapy (Supplementary Fig. S4C; Supplementary Table S4)], we also observed results consistent with the overall cohort, with the LRT significant for interaction between IRS status and anti-PD-(L)1 versus previous therapy in each group.

In addition, as previous reports have suggested that a ratio of >1.3 for subsequent PFS compared with the immediately preceding line PFS (PFS2/PFS1) supports clinical benefit of the subsequent therapy, we also determined the PFS2/PFS1 ratio for IRS-H versus IRS-L patients in the case cross-over cohort. As shown in Supplementary Table S5, across the 77 evaluable patients in the overall case–control cohort, the rate of PFS2/PFS1 >1.3 was significantly greater in IRS-H versus IRS-L patients [13/18 (72%) vs. 14/59 (24%), Cochran–Mantel–Haenszel OR 6.6 (95% CI, 2.2–20.2), *P* = 0.0005]; despite the smaller number of IRS-H patients in the MSS/TMB-L patient subset, results were similar [3/4 (75%) vs. 14/59 (24%), Cochran–Mantel–Haenszel OR 4.4 (95% CI, 1.1–16.9), *P* = 0.03]. Taken together, these results confirm the predictive (vs. prognostic) nature of the IRS biomarker and demonstrate prolonged clinical benefit in IRS-H patients treated with PD-(L)1 in the >first-line setting.

### IRS versus PD-L1 IHC and TMB Alone for Predicting Anti-PD-L1 rwPFS Benefit

**FIGURE 2:**
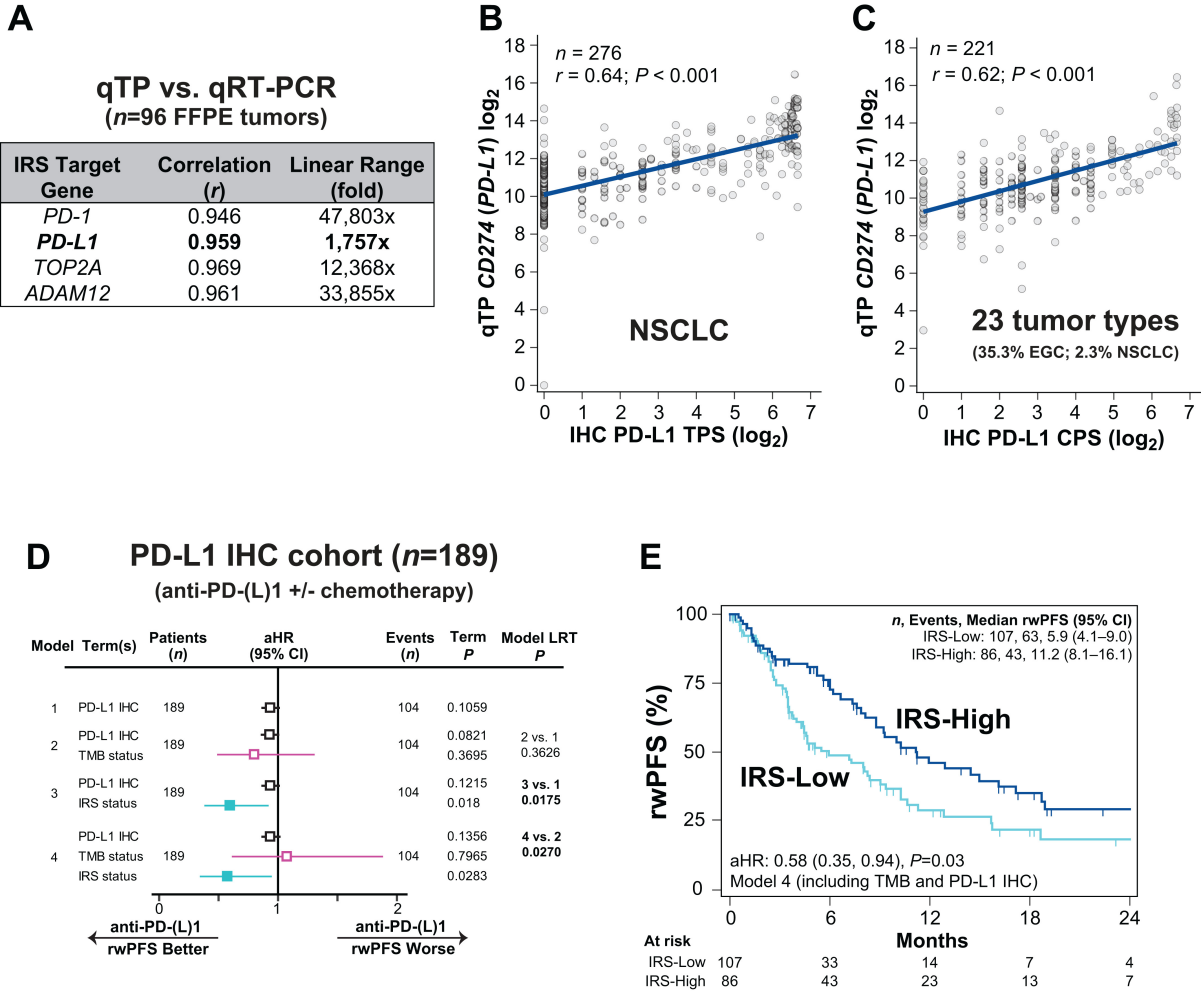
Confirmation of the added utility of the IRS versus clinical PD-L1 IHC and TMB alone. **A,** Normalized PD-L1 (*CD274*) expression (and the other IRS expression components) by the qTP platform used to generate IRS were validated versus qRT-PCR in a validation cohort of 96 FFPE tumor tissue samples tested by clinical CGP and in parallel qTP. The Pearson correlation and linear range of each component is shown (Supplementary Fig. S1). **B** and **C**, The Pearson correlation of normalized *PD-L1* expression by qTP [log_2_ normalized reads per million (nRPM) units] versus clinical PD-L1 IHC score (in submitted pathology reports) was determined in two cohorts of clinical FFPE tumor tissues [regardless of TMB availability and anti-PD-(L)1 treatment]. **B,** PD-L1 expression by qTP (log_2_ normalized units) versus PD-L1 IHC by TPS [using the 22C3 antibody clone (log_2_ TPS) in 276 clinically tested FFPE NSCLC tumors with available TPS is plotted]. The linear fit, Pearson correlation (*r*), and *P* value are shown. **C,** PD-L1 expression by qTP versus PD-L1 IHC by CPS using the 22C3 antibody clone (log_2_ CPS) in 221 clinically tested FFPE tumors (23 tumor types; most frequently EGC) with available TPS is plotted. The linear fit, Pearson correlation (*r*), and *P* value are shown. **D,** Using B and C, we identified a cohort of all 189 eligible NCT03061305 patients with IRS and PD-(L)1 IHC in accompanying pathology reports who were treated with anti-PD-(L)1 therapy (± chemotherapy). The association of biomarkers with anti-PD-(L)1 rwPFS was determined by Cox proportional hazards modeling [adjusting for age, sex assigned at birth, line of therapy, tumor type, therapy type (monotherapy vs. chemotherapy combination), and inclusion in IRS discovery status]. PD-L1 IHC score (continuous; log_2_) was included in the baseline model (Model 1), with the aHR, 95% CI, number (*n*) of patients and events, and *P* value shown for the biomarker term by forest plot. TMB status (-H vs. -L; pink) and IRS status (-H vs. -L; light blue) were separately added to this model (Models 2 and 3, respectively). The significance of each biomarker term is shown and the *P* value of the LRT comparing the full (Model 2 or 3) versus reduced (Model 1) model is shown. Model 4 includes PD-L1 IHC, TMB, and IRS. Significant biomarker terms are shown by filled in aHR estimates. **E,** Anti-PD-(L)1 rwPFS stratified by IRS group is shown by unadjusted Kaplan–Meier analysis, with the aHR from Model 4 in D shown. See Supplementary Table S7 for full subgroup analysis.

While CGP assessable biomarkers (TMB and MSI) are used largely to inform on >first-line anti-PD-(L)1 monotherapy treatment outside of approved indications, PD-L1 IHC is a companion or complementary diagnostic used to guide many first-line anti-PD-(L)1 monotherapy or combination therapy treatments. As IRS includes both TMB and *PD-L1* expression (by qTP), it is unclear whether the more quantitative nature of qTP and additional gene expression biomarkers in IRS (Supplementary Fig. S1; Fig. 2A) add clinical utility beyond TMB status and PD-L1 IHC alone. We previously showed that *PD-L1* expression by qTP was significantly associated with categorical PD-L1 IHC expression [0, 1%–49%, ≥50% by 22C3 tumor proportion scoring (TPS)] in pathology reports accompanying NSCLC tumors with valid qTP data (20); however, IRS status and/or anti-PD-(L)1 therapy outcome were not available for the majority of that cohort.

Hence, here, we first demonstrated that in both the previously reported NSCLC cohort, where PD-L1 IHC expression was evaluated by TPS (*n* = 276), and in a cohort of 221 tumors from 23 tumor types [most frequent EGC (35.3%); only 2.3% NSCLC] with accompanying PD-L1 IHC expression by 22C3 combined positive scoring (CPS), *PD-L1* qTP and IHC expression were only moderately correlated (Pearson *r* = 0.64 and *r* = 0.62, respectively; both *P* < 0.001; Fig. 2B and C), despite the highly correlated (Pearson *r* = 0.96 and 1,757x linear range) nature of *PD-L1* gene expression by qTP versus qRT-PCR (Fig. 2A). To identify a cohort to compare the clinical utility of IRS versus PD-L1 IHC and/or TMB, we identified all eligible NCT03061305 patients [including those in the discovery cohort (20) given the limited numbers] treated with anti-PD-(L)1 therapy (± chemotherapy) and available PD-L1 IHC (from above; *n* = 177 eligible), and identified and included an additional 12 eligible patients with PD-L1 IHC by SP142 [reported as tumor-infiltrating immune cell (IC) score] (Supplementary Fig. S2; Supplementary Methods S1).

Across this 189 patient PD-L1 IHC cohort (overall 10 tumor types; 36.0% non-NSCLC), only 1 patient (0.5%) was MSI-H (who was also TMB-H), 25.9% were TMB-H, and 43.9% were IRS-H (Supplementary Table S6); median total follow-up in these patients was 10.9 months, with 106 (56.1%) progression events. To compare utility of IRS with PD-L1 IHC and/or TMB alone, we first determined the association of PD-L1 IHC alone [reported IHC score; continuous, aHR 0.93 (95% CI, 0.86–1.01), *P* = 0.11] when controlling for relevant clinical variables in a Cox proportional hazards model in the 189 patients (Fig. 2D). Of note, continuous PD-L1 IHC resulted in a better overall model fit versus categorical PD-L1 IHC (<1 vs. ≥1) and was hence used in the baseline model.

Next, while TMB status alone was not a significant term when added to this model (nor did it improve model performance), IRS status was both a significant term [aHR 0.59 (95% CI, 0.39–0.91), *P* = 0.02] and significantly improved the PD-L1 IHC only model (LRT *P* = 0.02). Importantly, IRS status remained both a significant term [aHR 0.58 (95% CI, 0.35–0.94), *P* = 0.03] and significantly improved the model including both PD-L1 IHC and TMB status (LRT *P* = 0.03; Fig. 2D and E). Despite clear limitations of this cohort, including the inclusion of discovery cohort patients, sensitivity analysis demonstrated generally consistent results across subgroups (Supplementary Table S7), and these results support added clinical utility of the integrative IRS biomarker versus PD-L1 IHC and/or TMB (and MSI) alone for predicting anti-PD-(L)1 rwPFS.

### IRS is Robust Across Self-reported Racial Groups

**FIGURE 3:**
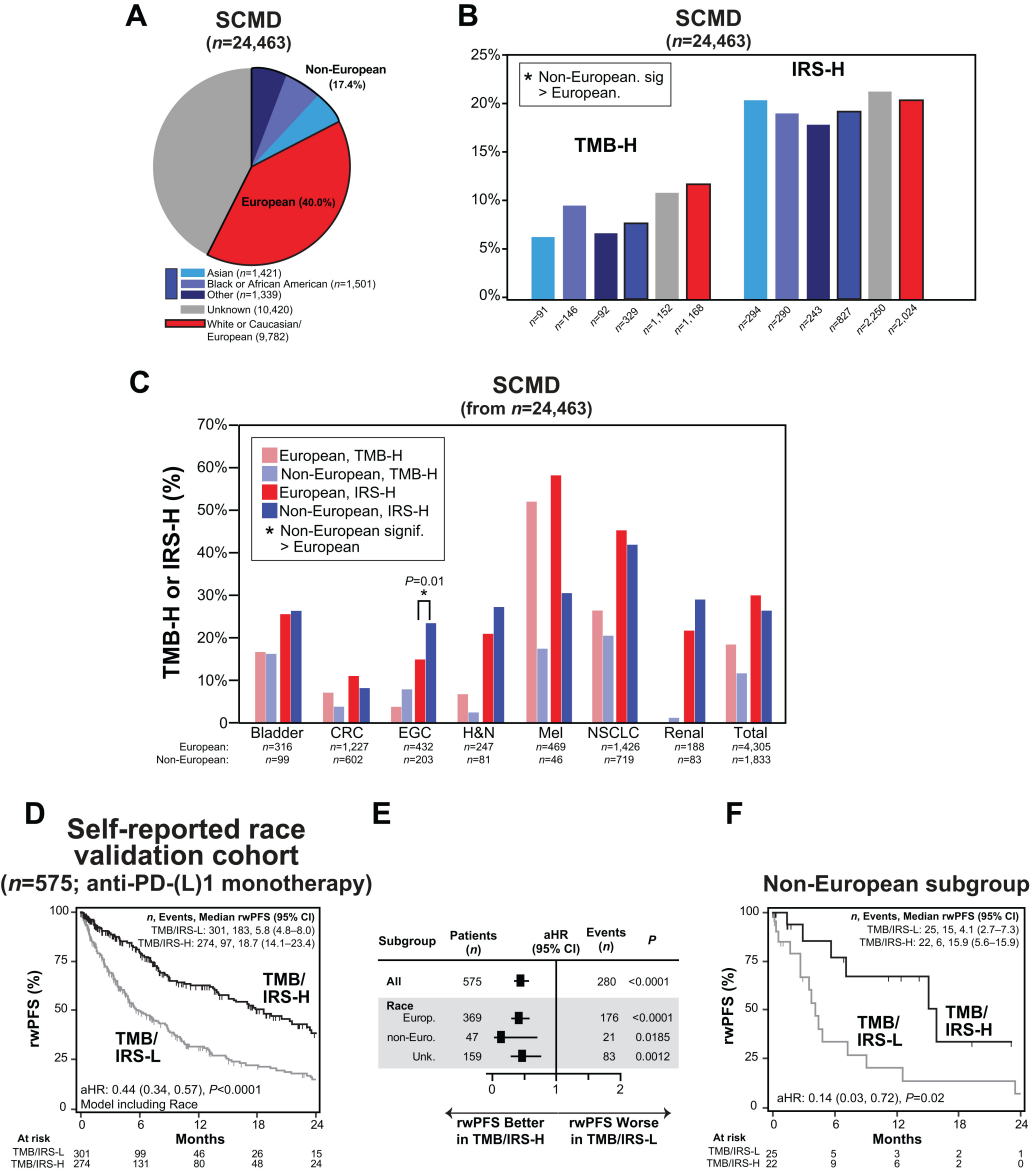
IRS is robust to self-reported race. **A,** Pie chart of self-reported race for all 24,463 patients in the SCMD with informative TMB and gene expression data needed to generate IRS regardless of treatment history (SCMD lock at the time of IRS development). The total number of patients in each racial group is shown. **B,** The percentage of TMB-H and IRS-H patients from the SCMD (*n* = 24,463 as in A stratified by self-reported race is plotted). Fisher exact test was used to test the differences in IRS-H (or TMB-H) between the White or Caucasian/European group and all other groups; groups where IRS-H (or TMB-H) was significantly greater (*P* < 0.05) versus the White or Caucasian group (potentially as a consequence of inappropriate filtering of germline variants in TMB determination for non-White or Caucasian groups) are indicated by *. Asian, Black or African American and Other groups were also considered together as non-European (blue). **C,** Further breakdown by TMB and IRS status and relevant tumor types. The percentage of IRS-H (bold hue) and TMB-H (light hue) stratified by White or Caucasian/European (red) and non-European (blue) self-reported race is plotted for the *n* = 6,138 total patients from A and B with one of the seven indicated primary tumor types (CRC = colorectal, EGC = esophagogastric, H&N = head and neck, Mel = melanoma, NSCLC = non–small cell lung carcinoma). The total number of European and non-European patients with each tumor type are indicated. **D,** Across eligible NCT03061305 patients treated with anti-PD-(L)1 monotherapy, we identified a validation cohort of all 575 patients not included in IRS discovery to assess the robustness of IRS (and the TMB component) to self-reported race. Anti-PD-(L)1 monotherapy rwPFS stratified by combined TMB and IRS status [TMB-H or IRS-H (TMB/IRS-H; black) vs. TMB-L and IRS-L (TMB/IRS-L; gray)] is shown (left) by unadjusted Kaplan–Meier analysis with the aHR [adjusted for age, sex assigned at birth, line of therapy, tumor type, anti-PD-(L)1 therapy type, inclusion in previous validation cohort, and self-reported race (non-European, unknown, or European)], 95% CI and *P* value for TMB/IRS status (TMB/IRS-H vs. TMB/IRS-L) shown. The number (*n*) of patients, events, and median rwPFS (with 95% CI) for each group are shown. **E,** Forest plot of rwPFS by IRS status in the cohort (all) and each self-reported racial group is shown. Significant associations are shown by filled in aHR estimates. **F,** Anti-PD-(L)1 monotherapy rwPFS stratified by TMB/IRS status is shown by unadjusted Kaplan–Meier analysis for the non-European subgroup (as in the overall cohort).

Recent reports have highlighted the potential for CGP-based tumor-only tests to overestimate TMB at clinically relevant thresholds, particularly in patients with non-European ancestry (8), when germline variants are misclassified as somatic due to lower representation of non-Europeans in publicly available databases of genetic variation. Hence, as the TMB component of Strata Select used to generate the TMB in IRS is tumor-only, we assessed IRS-H and TMB-H frequencies across racial groups in the overall SCMD (self-reported race is optionally collected at Strata Trial enrollment), as well as directly assessed IRS performance in non-European patients. First, as shown in Fig. 3A, across the 24,463 consecutive patients in the SCMD at the time of IRS development [regardless of treatment (20)], 17.4% and 40.0% of patients self-identified as non-European and White or Caucasian/European, respectively, with the remainder of unknown race (due to not reporting). As shown in Fig. 3B and Supplementary Table S8, neither TMB-H nor IRS-H frequencies were significantly higher in self-reporting non-European groups (individually or collectively), or those of unknown race, compared with those self-reporting as White or Caucasian/European. Likewise, when limiting results to the seven tumor types highlighted by Nassar and colleagues (8), where they observed significantly greater TMB-H frequency in four of seven tumor types for self-reported non-European versus European patients in the Dana-Farber Cancer Institute (DFCI) tumor-only CGP cohort (Supplementary Table S9), only one of seven tumor types had significantly higher frequency of TMB-H or IRS-H in the SCMD for non-European versus European patients (IRS-H in EGCs; Fig. 3C; Supplementary Table S9).

To directly evaluate the performance of IRS in non-European patients, we identified a self-reported race validation cohort consisting of all eligible NCT03061305 patients treated with PD-(L)1 monotherapy who were not used in IRS discovery (20); as MSI status has not been shown to be impacted by race/ancestry, while both TMB and IRS status could be impacted by artifactual inflated TMB, we excluded all MSI-H patients (Supplementary Fig. S2). Demographics and tumor types for the 575 included patients are shown in Supplementary Table S10. We then determined the association of TMB-H or IRS-H (TMB/IRS-H) versus TMB-L and IRS-L (TMB/IRS-L) status with PD-(L)1 monotherapy rwPFS using the same approach as in Fig. 1, except additionally including a term for inclusion in the previous IRS validation cohort and a self-reported race term [non-European, or unknown (vs. European as the reference)] in the Cox proportional hazards model. As shown in Fig. 3D, while TMB/IRS-H versus TMB/IRS-L status remained significantly associated with PD-(L)1 monotherapy rwPFS [aHR 0.44 (95% CI, 0.34–0.57)], self-reported race was not [non-European vs. European aHR 1.17 (95% CI, 0.73–1.88; *P* = 0.51); unknown versus European aHR 1.12 (95% CI, 0.94–1.59, *P* = 0.14)]. Finally, subgroup analysis specifically demonstrated that like in European and unknown race patients, among the 47 non-European patients (Supplementary Table S10), those TMB/IRS-H had significantly longer rwPFS than those TMB/IRS-L [median rwPFS 15.9 (95% CI, 5.6–15.9) vs. 4.1 (95% CI, 2.7–7.3) months, aHR 0.14 (95% CI, 0.03–0.71), *P* = 0.02; Fig. 3E and F]. Similar results were observed excluding patients in the previous IRS validation cohort (Supplementary Fig. S5). Taken together, these results support the applicability of IRS to both self-reported non-European and European populations.

### Validation of Three-Group IRS Status for Predicting Anti-PD-(L)1 and/or Chemotherapy Benefit

Although the above results support the utility of IRS for stratifying anti-PD-(L)1 monotherapy response, nearly all tumor types with monotherapy indications also have anti-PD-(L)1 combination indications. Given analyses demonstrating independent drug actions of the individual components of PD-(L)1 combination regimen components as described earlier (23), we sought to determine whether IRS status could stratify PD-(L)1 response well enough to identify those patients (i) so unlikely to benefit that they could be spared the additional toxicity of the PD-(L)1 component, and/or (ii) those so likely to benefit that they could be spared the additional toxicity of the chemotherapy component. Given the likely need to further stratify predicted lack of anti-PD-(L)1 benefit beyond the IRS-H versus IRS-L threshold, we used the previously described three-group IRS classification (20), where IRS-L is divided into IRS-UL [least benefit from anti-PD-(L)1 monotherapy] and IRS-IL groups. As shown in Supplementary Fig. S6, in the 352 patient anti-PD-(L)1 monotherapy validation cohort described above, IRS-UL had the numerically shortest median rwPFS [IRS-UL, IRS-IL, and IRS-H median rwPFS 3.5 (95% CI, 1.9–6.4), 4.3 (95% CI, 2.8–5.4), and 15.1 (95% CI, 8.5–21.3) months, respectively; IRS-H versus -IL aHR 0.36 (95% CI, 0.24–0.55), *P* < 0.0001; IRS-IL versus -UL aHR 0.82 (95% CI, 0.56–1.22), *P* = 0.34], and three-group IRS status was predictive of pembrolizumab versus prior therapy benefit in both the full case cross-over cohort and the MSS/TMB-L subset (likelihood ratio test *P* < 0.0001 and *P* = 0.03, respectively).

**FIGURE 4:**
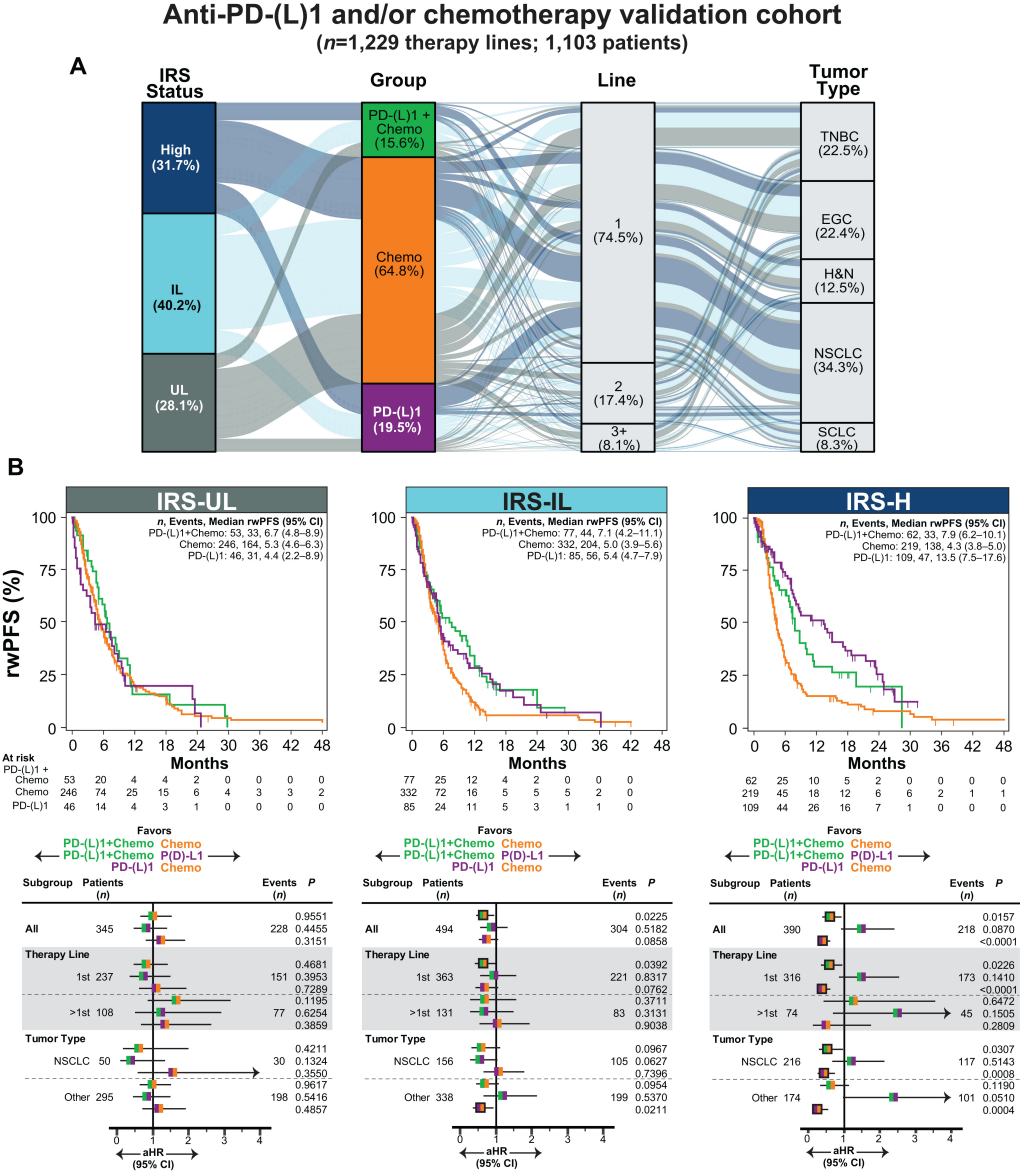
Validation of IRS to stratify anti-PD-(L)1, chemotherapy and anti-PD-(L)1 + chemotherapy benefit in relevant tumor types. **A,** Clinical characteristics of the anti-PD-(L)1 and/or chemotherapy (chemo) validation cohort are shown in an alluvial diagram. Across all eligible NCT03061305 patients treated with anti-PD-(L)1 monotherapy, chemotherapy, or anti-PD-(L)1 + chemotherapy, we identified a validation cohort of 1,229 total eligible therapy lines (from 1,103 patients) in five relevant tumor types with anti-PD-(L)1 and/or chemotherapy treatment decisions: NSCLC, TNBC, EGC, H&N, and SCLC. Anti-PD-(L)1 ± chemotherapy lines used in IRS training were excluded. The IRS model and three-group classification thresholds were used to assign IRS-UL (gray), IRS-IL (light blue), and IRS-H (dark blue) status. For all 1,229 eligible therapy lines, IRS status, treatment group [PD-(L)1: purple; chemo: orange; PD-(L)1+chemo green], the systemic line of treatment, and tumor types are shown. Stratum are colored by IRS status. **B,** rwPFS by treatment group is shown separately for each IRS group by unadjusted Kaplan–Meier analysis. The number (*n*) of patients, events, and median rwPFS (with 95% CI) are shown. Treatment group outcomes were compared in each IRS group by Cox proportional hazards modeling (adjusting for age, gender, treatment group, line of therapy, tumor type, and *PD-L1* RNA expression). Forest plots were used to visualize the aHR for each treatment group comparison, with the 95% CI, number of patients and events, and *P* value for each comparison shown. aHR estimates are colored by the treatment group comparison and significant associations are shown by outlined aHR estimates. In addition to the entire cohort (All), key subgroups are shown. See Supplementary Fig. S6 for covariate adjusted plots, Supplementary Fig. S7 for overlap weighting propensity score analysis, and Supplementary Table S12 for full subgroup analysis.

Hence, we identified all 1,103 eligible NCT03061305 patients [with 1,229 total chemotherapy, anti-PD-(L)1, or chemotherapy + anti-PD-(L)1 therapy lines] from the SCMD who were in one of five relevant tumor types: NSCLC, H&N, EGC, SCLC, and TNBC (Supplementary Fig. S2; Fig. 4A); anti-PD-(L)1 (with or without chemotherapy) lines used in IRS discovery or validation were specifically excluded. As shown in Fig. 4A, across the 1,229 lines, 240 (19.5%), 797 (64.8%), and 192 (15.6%) were anti-PD-L1 monotherapy, chemotherapy, and anti-PD-(L)1 + chemotherapy, respectively, with NSCLC being the most frequent tumor type (34.3%); median total follow-up was 11.9 months, with 750 (61.0%) progression events. Additional clinical data on this cohort are provided in Supplementary Table S11.

We then applied the three-group IRS classification scheme to this cohort, where across the 1,229 included lines, 345 (28.1%), 494 (40.2%), and 390 (31.7%) were in IRS-UL, IRS-IL, and IRS-H patients, respectively (Fig. 4A), and we compared group outcomes by Kaplan–Meier analysis and Cox proportional hazards modeling (unadjusted plots shown in Fig. 4B and covariate adjusted plots shown in Supplementary Fig. S7), including controlling for *PD-L1* RNA expression (given limited clinical PD-L1 IHC availability).

In the 345 IRS-UL patients, no significant differences between anti-PD-L1, chemotherapy, and anti-PD-(L)1 + chemotherapy rwPFS were observed (aHR 0.82–1.24, *P* = 0.32–0.96, Fig. 4B; Supplementary Table S12). In those IRS-IL (*n* = 494), anti-PD-(L)1 + chemotherapy rwPFS was significantly longer than chemotherapy rwPFS [aHR 0.66 (95% CI, 0.46–0.94), *P* = 0.02], while anti-PD-(L)1 had numerically longer rwPFS than chemo treatment [aHR 0.75 (95% CI, 0.54–1.04), *P* = 0.09]. While rwPFS did not significantly differ in IRS-IL patients treated with anti-PD-(L)1 versus anti-PD-(L)1 + chemotherapy [aHR 0.88 (95% CI, 0.56–1.31), *P* = 0.52], subgroup analysis showed that in patients with NSCLC, anti-PD-(L)1 + chemotherapy had numerically longer rwPFS versus both chemotherapy [aHR 0.61 (95% CI, 0.34–1.09), *P* = 0.10] and anti-PD-(L)1 [aHR 0.56 (95% CI, 0.30–1.03), *P* = 0.06]. Finally, in IRS-H patients (*n* = 390), anti-PD-(L)1 and anti-PD-(L)1 + chemotherapy rwPFS were both significantly longer than chemo rwPFS [aHR 0.41 (95% CI, 0.28–0.59), *P* < 0.0001 and aHR 0.61 (95% CI, 0.41–0.91), *P* = 0.02, respectively], while no significant difference in rwPFS was observed between anti-PD-(L)1 ± chemotherapy [aHR 1.5 (95% CI, 0.94–2.40), *P* = 0.09]. Sensitivity analysis demonstrated that in IRS-H patients, anti-PD-(L)1 + chemotherapy versus PD-(L)1 rwPFS were more similar in NSCLC [aHR 1.21 (95% CI, 0.69–2.12), *P* = 0.51] versus other tumor types [aHR 2.34 (95% CI, 0.996–5.71), *P* = 0.051], with the non-NSCLC analysis limited by the smaller number of PD-(L)1 treatments, consistent with the earlier line anti-PD-(L)1 + chemotherapy indications [vs. PD-(L)1 monotherapy] and lower IRS-H rates (Fig. 3C; ref. 20) in these tumor types versus NSCLC. Importantly, similar results were observed in the overall cohort using overlap weighting-based propensity score analysis (Supplementary Fig. S8), and sensitivity analyses demonstrated generally similar results across subgroups (Fig. 4B; Supplementary Table S12), including in NSCLC versus other tumor types, as well as in first or >first-line treatments, despite the limitations of this cohort representing real-world treatment patterns. Taken together, these results support additional utility for IRS status when -L is stratified to -IL and -UL groups, which inform on the comparative benefit of anti-PD-(L)1 monotherapy, chemotherapy, and anti-PD-(L)1 + chemotherapy.

## Discussion

Herein, leveraging clinical and molecular information from an ongoing observational clinical trial (NCT03061305), we first confirmed the performance and predictive nature of IRS, an integrative biomarker combining TMB and quantitative gene expression, to predict anti-PD-(L)1 monotherapy benefit by both rwPFS and OS in an independent validation cohort of 352 patients from 31 solid tumor types treated with PD-(L)1 monotherapy. In addition, we demonstrated that IRS-H versus -L status improves upon both currently available CGP biomarkers (TMB and MSI) as well as clinical PD-L1 IHC, for anti-PD-(L)1 benefit prediction. Importantly, through multiple analyses, we confirmed that IRS is robust across self-reported racial groups, an important consideration given the potential for the tumor only TMB component of IRS (and TMB as reported by the CGP test) to be impacted to by inappropriate classification of genetic variants as somatic (8). Finally, we confirmed utility for three group IRS status [where -L is divided into those least likely to benefit (-UL) and an intermediate group (-IL)] in guiding anti-PD-(L)1 and/or chemotherapy treatment decisions through an analysis of 1,229 treatment lines in five relevant tumor types.

In the anti-PD-(L)1 monotherapy validation cohort, IRS-H status was associated with significantly longer PD-(L)1 rwPFS (aHR 0.41, *P* < 0.0001) and OS (aHR 0.47, *P* = 0.0002) when adjusted for clinical covariates. Sensitivity analyses confirmed the association of IRS status with rwPFS and OS in key subgroups, including specifically those treated with pembrolizumab, as IRS was trained in pembrolizumab-treated patients and validated initially in those treated with other anti-PD-(L)1 monotherapy (20). In addition, beyond confirming applicability across tumor types, we confirmed the added clinical utility of IRS status to currently available pan-tumor biomarkers (MSI and TMB) through multiple approaches. We also confirmed the predictive (vs. prognostic) nature of IRS through a case cross-over analysis of patients treated with ≥second-line PD-(L)1, demonstrating both a significant interaction between treatment [anti-PD-(L)1 therapy vs. immediately preceding systemic therapy] and IRS status, as well as a significantly greater rate of rwPFS2/rwPFS1 >1.3, a ratio indicative of clinical benefit (25–27), in IRS-H versus IRS-L patients. Taken together, these results confirm the predictive utility of IRS for identifying patients likely to benefit from PD-(L)1 monotherapy, now specifically including pembrolizumab, beyond currently utilized pan-solid tumor biomarkers.

Outside of colorectal cancer, CGP assessable immunotherapy-related biomarkers (MSI and TMB) support second- or later-line treatment indications. In contrast, many tumor types use PD-L1 IHC to guide first-line treatment decisions for anti-PD-(L)1 therapy, alone or combined with chemotherapy, particularly in tumor types where immunotherapy is not the standard of care for all patients. Importantly however, multiple IHC assays and scoring systems are used across tumor types (9–19), and a pan-cancer diagnostic IHC approach has not been advanced. In addition, interpretation of clinically relevant cutoffs show substantial variability in real world practice, particularly using the CPS scoring system at lower cutoffs, as demonstrated in a recent study of the companion diagnostic PD-L1 for gastric cancer (22C3 PD-L1 IHC with the CPS system), where Fernandez and colleagues showed an overall percent agreement of only 30% using the approved CPS <1 versus ≥1 threshold when 14 pathologists evaluated the same IHC slide (28). Hence, it is not surprising that while the *PD-L1* RNA expression component of IRS was highly correlated versus qRT-PCR (Pearson *r* = 0.96 and >1,757x fold linear range), correlation with PD-L1 IHC by TPS and CPS was modest (Pearson *r* = 0.64 and 0.62, respectively). Most importantly, herein, using a cohort of 189 tumors from 10 tumor types (where PD-L1 IHC by TPS, CPS, or IC was ordered clinically), IRS status outperformed PD-L1 IHC, or combined PD-L1 IHC and TMB status, for predicting anti-PD-(L)1 rwPFS. Although this cohort had several limitations, including being comprised of both discovery and validation cohort patients (controlled in the CPH model) and having near-exclusively positive CPS/IC results [as expected given such results guide anti-PD-(L)1 therapy in current practice], trends were consistent across subgroups, and these results support known issues with PD-L1 IHC in routine practice. Taken together, this analysis demonstrates the utility of the integrative IRS biomarker beyond PD-L1 IHC and CGP assessable biomarkers alone.

TMB has been shown to be a predictive biomarker for pembrolizumab monotherapy benefit across tumor types analyzed in the KEYNOTE clinical trials through both tumor-only CGP sequencing (4) as well as whole-exome sequencing (WES) with matched normal tissue (5), both of which supported the pan-solid tumor approval of pembrolizumab monotherapy for TMB-H tumors in the >first line (29). Unlike simple biomarkers such as BRAF p.V600E mutation detection for predicting BRAF/MEK inhibition in melanoma and other tumor types, TMB has numerous potential challenges as a predictive biomarker, including the potential for tumor-only CGP to overestimate TMB (and TMB-H rates) due to the inappropriate inclusion of true germline variants as false-positive somatic mutations. Most recently, Nassar and colleagues demonstrated through analyzing tumor-only CGP (from internal DFCI clinical testing) and paired-tumor/normal CGP (from MSK-IMPACT) and WES (The Cancer Genome Atlas), that tumor-only CGP particularly overestimated TMB and impacted the predictive nature of the biomarker in patients with non-European ancestry, as such groups are less represented in population reference databases (6, 7), questioning the trans-ethnic applicability of TMB (8). Herein, we show that both TMB-H [by StrataNGS tumor-only CGP (24)] and IRS-H [which includes that TMB (20)], show little to no evidence of inflation in self-identified non-European populations across the entire SCMD population. In addition, we directly showed that TMB-H or IRS-H status (TMB/IRS-H) is associated with significantly longer PD-(L)1 rwPFS in self-identified non-Europeans, as well as other racial groups.

The filtering of candidate mutations for the TMB point estimate for Strata Select (24), which is both reported directly and used for IRS, has very low population frequency limits (any mutation reported in gnomAD is excluded), excludes mutations with variant allele frequency (VAF) between 45% and 55% in samples with a final molecularly informed tumor content (MTC) <80% (such variants are however retained for calculating the upper bound of the TMB estimate CI), and only includes mutations with VAF >one-fourth of the MTC [given that clonal mutations and tumor content correction have consistently been reported to better predict PD-(L)1 benefit than all mutations (30, 31)]. The combination of these three filtering steps helps to remove false-positive germline mutations regardless of their relative frequency in population databases, with the latter step additionally removing germline variants in regions of LOH in high MTC samples (when the germline variant is on the lost allele).

Taken together, these results show how TMB is more test specific than qualitative CGP biomarkers (e.g., detection or absence of *KRAS* mutations), and highlight the challenges of efforts to “harmonize” TMB estimates across testing platforms (5, 30, 32–35), arguing instead for the direct demonstration of clinical validity for predicting PD-(L)1 monotherapy benefit (across both European and non-European populations), as demonstrated previously (20) and confirmed herein for IRS.

As described above, unlike PD-L1 IHC, CGP assessable biomarkers (MSI and TMB) are largely useful in practice to guide anti-PD-(L)1 monotherapy outside of approved tumor types in patients with later-line tumors. Furthermore, beyond the most responsive tumor types (e.g., ultraviolet radiation–driven tumors), first-line anti-PD-L1 treatments have largely been advanced in combination with other agents (most commonly chemotherapy) given the modest response rates observed to anti-PD-(L)1 monotherapy in most tumor types (36). Although synergy between anti-PD-(L)1 combination regimen components has been proposed, such an effect has not been observed to date in approved combinations (1, 21–23), supporting the potential of sufficiently accurate anti-PD-(L)1 therapy biomarkers to both identify those patients who are unlikely to benefit from the addition of anti-PD-(L)1 therapy and identify those patients who are likely to benefit from anti-PD-(L)1 therapy alone. Given the generalizable nature of IRS for predicting anti-PD-L1 monotherapy across tumor types as validated herein, we also evaluated the utility of IRS for guiding anti-PD-(L)1 monotherapy versus combination (with chemotherapy) in five relevant tumor types (NSCLC, TNBC, EGC, H&N, and SCLC) where we also had sufficient chemotherapy alone treatments to confirm the predictive nature of IRS. For this use case, we used the previously described three-group IRS classification [where IRS-Low is divided into groups with intermediate (IL) and ultralow (UL) benefit as shown in IRS development (20)] given the need to identify the most and least responsive anti-PD-(L)1 populations. Importantly, as shown in this validation cohort, IRS-UL patients showed no significant benefit of anti-PD-(L)1 combined with chemotherapy versus chemotherapy alone, while IRS-H patients showed no significant benefit of anti-PD-(L)1 combined with chemotherapy versus anti-PD-(L)1 alone. This cohort has several notable limitations, most importantly that it used rwPFS (OS is impacted by later line therapies), it reflects real-world treatments often guided by PD-L1 IHC, and several included tumor types do not have both anti-PD-(L)1 monotherapy and combination chemotherapy indicated/recommended in the same treatment line. However, sensitivity analyses demonstrated consistent trends in key subgroups, including patients treated in the first or >first line, as well as in patients with NSCLC (confirming our previous findings) or other tumor types. Likewise, both use cases of IRS [identifying those patients unlikely to benefit from anti-PD-(L)1 added to chemotherapy as well as those unlikely to benefit from chemotherapy added to anti-PD-(L)1] are well supported conceptually beyond the independent drug actions observed in pivotal trials and the challenges in interpreting PD-L1 IHC described above (23, 28). For example, based on the pivotal first-line trials (37, 38), current NCCN guidelines (v2.2023) consider chemotherapy alone, chemotherapy combined with nivolumab (category 1 if CPS> = 10; category 2B if <10), and chemotherapy combined with pembrolizumab [category 1 or 2A if CPS > 10 (based on the specific chemotherapy); category 2B if <10] each as preferred first line regimens for advanced esophageal adenocarcinoma, highlighting the need for additional biomarkers to guide this treatment decision. Likewise, although pembrolizumab with or without chemotherapy have not been compared directly in the most anti-PD-(L)1 responsive subset of patients with nonsquamous NSCLC (those with TPS ≥ 50%), concurrent 5-year updates of both pivotal trials (KEYNOTE-042 and KEYNOTE-189) showed similar OS HRs of the pembrolizumab containing versus chemotherapy only arms [pembrolizumab only: 0.68 (0.57--0.81); pembrolizumab + chemotherapy: 0.68 (0.49–0.96; refs. 39, 40)], arguing strongly against substantial synergy between chemotherapy and pembrolizumab in this indication (23). Taken together, these results support the clinical utility of IRS as a useful tool to help guide chemotherapy versus anti-PD-(L)1 monotherapy versus anti-PD-(L)1 + chemotherapy decision making, the most relevant “precision medicine” opportunity for many patients with advanced cancer.

As a study on real-world treatment outcomes, the major limitation is that cohorts analyzed herein reflect usage of PD-(L)1 in real-world practice. In the monotherapy validation cohort, IRS-H patients not indicated for anti-PD-(L)1 treatment (whether by tumor type, MSI-H or TMB-H) are poorly represented, as would be expected from a real-world cohort where IRS status was not provided to guide treatment. In addition, clinical prognostic factors (e.g., performance status, disease burden, tobacco exposure history, and blood biomarkers such as lactate dehydrogenase) are not collected as part of NCT03061305, and hence were not available for inclusion in outcome models. Nevertheless, this study demonstrated that IRS-H versus -L status robustly stratified anti-PD-(L)1 monotherapy benefit (by both rwPFS and OS) across tumor types, IRS status was predictive in those treated with anti-PD-(L)1 in the >first line, and IRS added benefit beyond MSI/TMB status alone. These results confirm those observed in the initial IRS validation that included 248 patients (from 24 tumor types) treated with non-pembrolizumab anti-PD-(L)1 monotherapy (20). Like the analysis of KEYNOTE studies by CGP (4) and WES (5) leading to the pan-solid tumor approval of pembrolizumab in the >first-line setting (29), which did not assess all possible tumor types but observed relatively consistent pembrolizumab monotherapy benefit in TMB-H patients across tumor types, results from our current study further support the pan-solid tumor predictive nature of IRS-H status for anti-PD-(L)1 monotherapy benefit (20). In addition, given the limited number of MSI/TMB-H patients who are IRS-L, their outcome is unclear, and clinically treatment associations are made based on MSI/TMB-H status given the level 1 treatment associations with these established biomarkers. As described above, our PD-L1 IHC cohort had several limitations. Ideally, such an analysis would be performed in patients randomized to receive anti-PD-(L)1 therapy versus control, regardless of PD-L1 IHC results; however, such cohorts are not readily accessible, although efforts to evaluate IRS in such scenarios are ongoing. Although we used self-reported race instead of genetic ancestry to assess TMB and IRS in European and non-European populations, TMB-H estimates were similar in the Nassar and colleagues DFCI cohort (8) using either approach (Supplementary Table S6), suggesting that this is unlikely to confound our results. While the non-European cohort assessed herein is small (*n* = 47), TMB/IRS-H status was significantly associated with anti-PD-(L)1 rwPFS in this population (both in the combined validation cohort and the subgroup excluding patients from the previous IRS validation) and the similarity of IRS-H (and TMB-H) rates in both European and non-European populations across the entire 23,643 patient SCMD population support the robustness of IRS to all populations. Like the PD-L1 IHC cohort, our anti-PD-(L)1 and/or chemotherapy validation cohort analysis would ideally be performed in patients randomized to receive anti-PD-(L)1, chemotherapy, or anti-PD-(L)1 + chemotherapy regardless of biomarker testing, however as pointed out above, such trials are either not readily accessible or have not been performed. Likewise, sufficient patients were not available to assess non-chemotherapy combinations (most relevant in melanoma, renal cell carcinoma, endometrial carcinoma, and most recently bladder cancer). Such use cases may also require development of integrative signatures that predict benefit from the non-anti-PD-(L)1 therapy components. The qTP platform used to report IRS is targeted, which was not a limitation during IRS development (given the substantial body of translational research addressing this use case); however, more discovery-based approaches—such as whole-transcriptome RNA sequencing—may be needed to inform candidates for additional expression signatures, including predicting non-chemotherapy agent benefit.

Here we demonstrate that IRS is predictive of anti-PD-(L)1 monotherapy rwPFS and OS in a validation cohort of 352 patients from 25 tumor types and added utility to MSI and TMB alone. In additional cohorts, we demonstrated that PD-L1 IHC and TMB were not significantly associated with anti-PD-(L)1 rwPFS when included in a model with IRS status. IRS (and the TMB component) were also shown to be robust in both European and non-European populations. Finally, we applied three-group IRS status (IRS-UL, IRS-IL, and IRS-H) to a 1,229-line validation cohort from five relevant tumor types, where we showed that rwPFS did not significantly differ between anti-PD-(L)1 + chemotherapy versus chemotherapy alone in IRS-UL patients, and rwPFS did not significantly differ between anti-PD-(L)1 + chemotherapy vs. anti-PD-(L)1 alone in IRS-H patients. Taken together, these results validate and extend the clinical utility of IRS—which is now covered for Medicare beneficiaries as part of the combined CGP + qTP Strata Select test—for guiding both anti-PD-(L)1 and/or chemotherapy treatment decisions.

## Supplementary Material

Supplementary Figure S1 Supplementary Figure S1 shows analytical validation of the gene expression component of Immunotherapy Response Score components vs. qRT-PCR

Supplementary Figure S2 Supplementary Figure S2 shows a diagram of the overall study

Supplementary Figure S3 Supplementary Figure S3 shows how Immunotherapy Response Score adds to tumor mutation burden and microsatellite instability status for anti-PD-(L)1 monotherapy clinical benefit

Supplementary Figure S4 Supplementary Figure S4 shows a validation of the predictive nature of Immunotherapy Response Score

Supplementary Figure S5 Supplementary Figure S5 shows robustness of Immunotherapy Response Score to self-reported race

Supplementary Figure S6 Supplementary Figure S6 shows three group Immunotherapy Response Score classification of the anti-PD-(L)1 monotherapy validation cohort

Supplementary Figure S7 Supplementary Figure S7. shows covariate adjusted Kaplan-Meier analysis of the chemotherapy, anti-PD-(L)1, and chemotherapy + anti-PD-(L)1 validation cohort

Supplementary Figure S8 Supplementary Figure S8. shows overlap weighting propensity score analysis of the chemotherapy, anti-PD-(L)1, and chemotherapy + anti-PD-(L)1 validation cohort

Supplementary Methods S1 Supplementary Methods S1 shows the additional methods and references supporting the main manuscript

Supplementary Table S1 Supplementary Table S1 shows the characteristics of the anti-PD-(L)1 monotherapy cohort

Supplementary Table S2 Supplementary Table S2 shows the adjusted Cox proportional hazards models for real-world progression free survival and overall survival in the monotherapy validation cohort

Supplementary Table S3 Supplementary Table S3 shows unadjusted restricted mean survival time analysis of for real-world PFS and overall survival in the monotherapy validation cohort

Supplementary Table S4 Supplementary Table S4 shows the characteristics of the patients used in the case cross-over analysis

Supplementary Table S5 Supplementary Table S5 shows rates of rwPFS2/rwPFS1 in the case cross-over analysis

Supplementary Table S6 Supplementary Table S6 shows the characteristics of the PD-L1 IHC cohort

Supplementary Table S7 Supplementary Table S7 shows a sub-group analysis of the PD-L1 IHC cohort

Supplementary Table S8 Supplementary Table S8 shows tumor mutation burden and Immunotherapy Response Score status by self -reported race in the Strata Clinical Molecular Database

Supplementary Table S9 Supplementary Table S9 shows tumor mutation burden and Immunotherapy Response Score status by self -reported race in relevant tumor types from the Strata Clinical Molecular Database compared to Nasser et al.

Supplementary Table S10 Supplementary Table S10 shows the characteristics of the anti-PD-(L)1 monotherapy self-reported race validation cohort and the non-European subset

Supplementary Table S11 Supplementary Table S11 shows the characteristics of the chemotherapy, anti-PD-(L)1, and chemotherapy + anti-PD-(L)1 validation cohort

Supplementary Table S12 Supplementary Table S12 shows a sub-group analysis of the chemotherapy, anti-PD-(L)1, and chemotherapy + anti-PD-(L)1 validation cohort

Supplementary Data S1 Supplementary Data S1 shows the REMARK checklist for the study

Supplementary Data S2 Supplementary Data S2 shows the clinical and molecular data for the monotherapy validation cohort
