# Supplementary material for: Validation of Immunotherapy Response Score as Predictive of Pan-solid Tumor Anti-PD-1/PD-L1 Benefit
Source: Cancer Res Commun. 2023 Jul 25;3(7):1335–49. doi: 10.1158/2767-9764.CRC-23-0036 (PMC10367935; doi:10.1158/2767-9764.CRC-23-0036)
Supplement: Supplementary Figure S1 — shows analytical validation of the gene expression component of Immunotherapy Response Score components vs. qRT-PCR [file crc-23-0036-s01.pdf]

Supplementary Figure S1

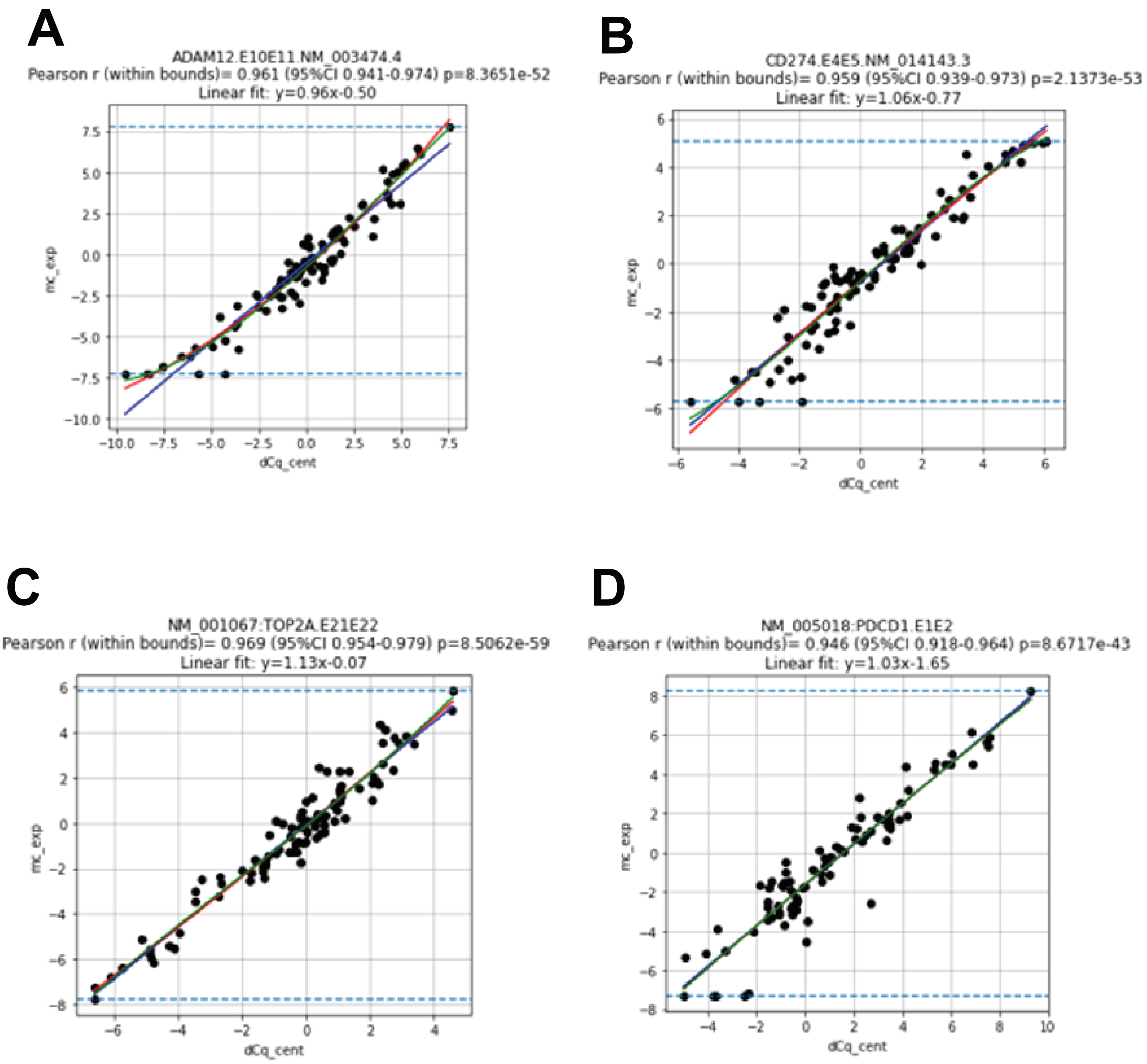

IRS Target	Within LOQ bound Pearson correlation coefficient (*r*)	Lower LOQ (l2nRPM)	Upper LOQ (l2nRPM)	Linear Range (fold)
ADAM12	0.961	-7.26	7.79	33,855x
PD-L1	0.959	-5.72	5.05	1,757x
TOP2A	0.969	-7.76	5.83	12,368x
PD-1	0.946	-7.30	8.24	47,803x

Supplementary Figure S1. Accuracy and linearity of Immunotherapy Response Score (IRS) expression components by quantitative transcriptional profiling vs. qRT-PCR

A-D. Normalized target gene expression for all four expression components of IRS (*ADAM12* [**A**], *PD-L1* [*CD274*; **B**], *TOP2A* [**C**] and *PD-1* [*PDCD1*; **D**] were determined by quantitative transcriptional profiling (performed in parallel with clinical StrataNGS CGP testing; y axis [mc_exp]) and qRT-PCR (x axis [dCq_cent]) from replicate RNA aliquots of 96 FFPE tumor tissues. Median-centered $\log_2$ values for each target gene are plotted. Blue, red, and green lines are linear (1st), 2nd order, and 3rd order models, respectively. Polynomial terms were not significant (all $p>0.05$) for any of the genes. Blue dashed lines are lower and upper limit of quantification (LOQ). The amplicon name, within LOQ bound Pearson correlation coefficient (*r*) with 95% confidence interval (CI), p-value, and linear fit equation are shown for each IRS expression component gene. The table below the individual plots shows the linear range (from lower to upper LOQ) for each gene.
