# Supplementary material for: Validation of Immunotherapy Response Score as Predictive of Pan-solid Tumor Anti-PD-1/PD-L1 Benefit
Source: Cancer Res Commun. 2023 Jul 25;3(7):1335–49. doi: 10.1158/2767-9764.CRC-23-0036 (PMC10367935; doi:10.1158/2767-9764.CRC-23-0036)
Supplement: Supplementary Figure S2 — shows a diagram of the overall study [file crc-23-0036-s02.pdf]

Supplementary Figure S2

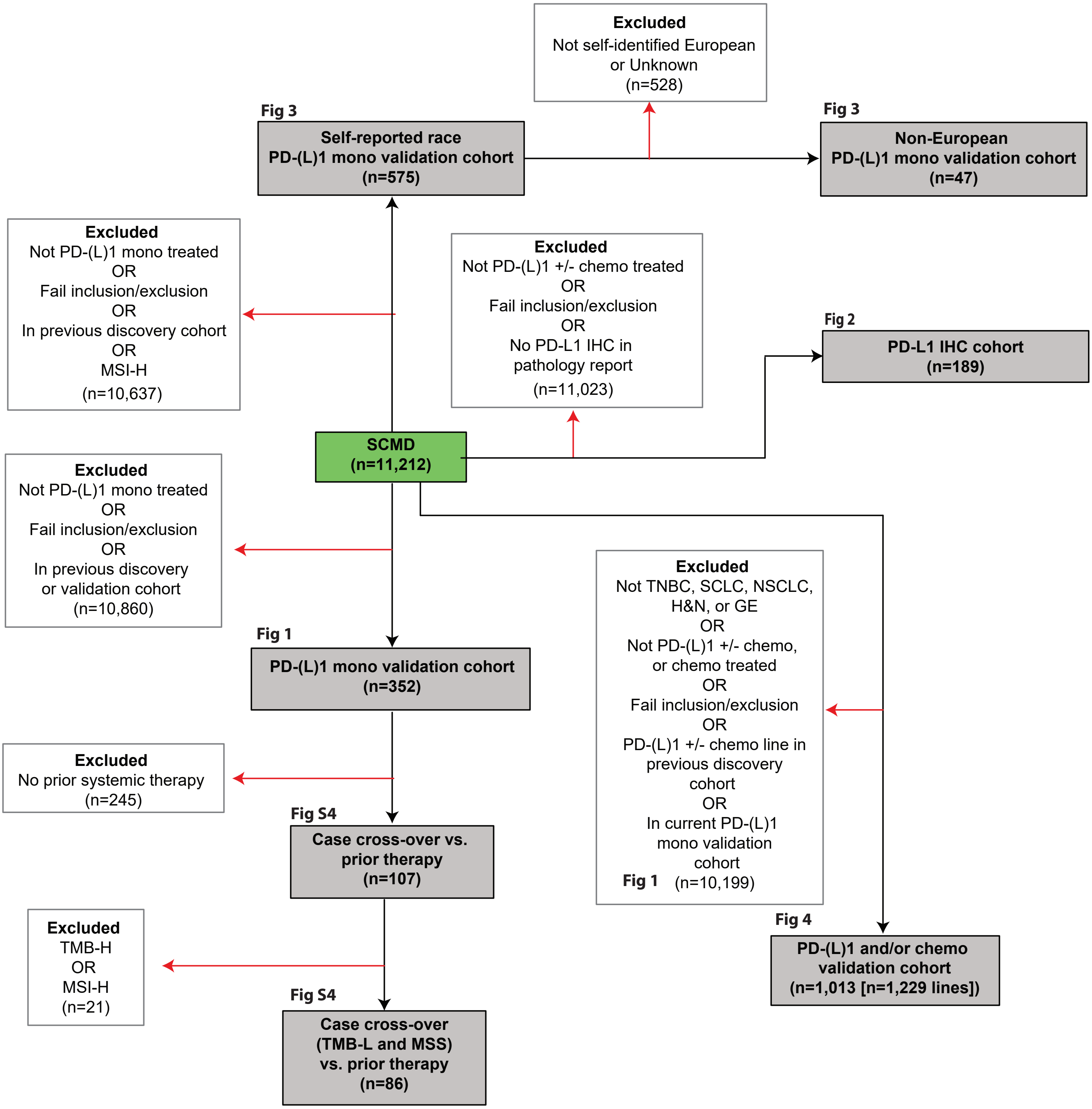

Supplementary Figure S2. Overall study diagram

Disposition of patients from the Strata Trial (NCT03061305) with clinical and molecular data in the Strata Clinical Molecular Database (SCMD) used herein to validate IRS in across clinically relevant cohorts (anti-PD-[L]1 monotherapy [mono] validation cohort, **Fig 1**; PD-L1 IHC cohort, **Fig 2**; self-reported race anti-PD-[L]1 mono validation cohort, **Fig 3**; anti-PD-(L) and/or chemotherapy [chemo] validation cohort, **Fig 4**) shown. Included populations (with the number of included patients) are indicated by gray boxes; excluded populations and reasons for exclusion are shown in white boxes. Patients can be shared across the four cohorts but are not indicated.
