# Supplementary material for: Validation of Immunotherapy Response Score as Predictive of Pan-solid Tumor Anti-PD-1/PD-L1 Benefit
Source: Cancer Res Commun. 2023 Jul 25;3(7):1335–49. doi: 10.1158/2767-9764.CRC-23-0036 (PMC10367935; doi:10.1158/2767-9764.CRC-23-0036)
Supplement: Supplementary Figure S3 — shows how Immunotherapy Response Score adds to tumor mutation burden and microsatellite instability status for anti-PD-(L)1 monotherapy clinical benefit [file crc-23-0036-s03.pdf]

Supplementary Figure S3

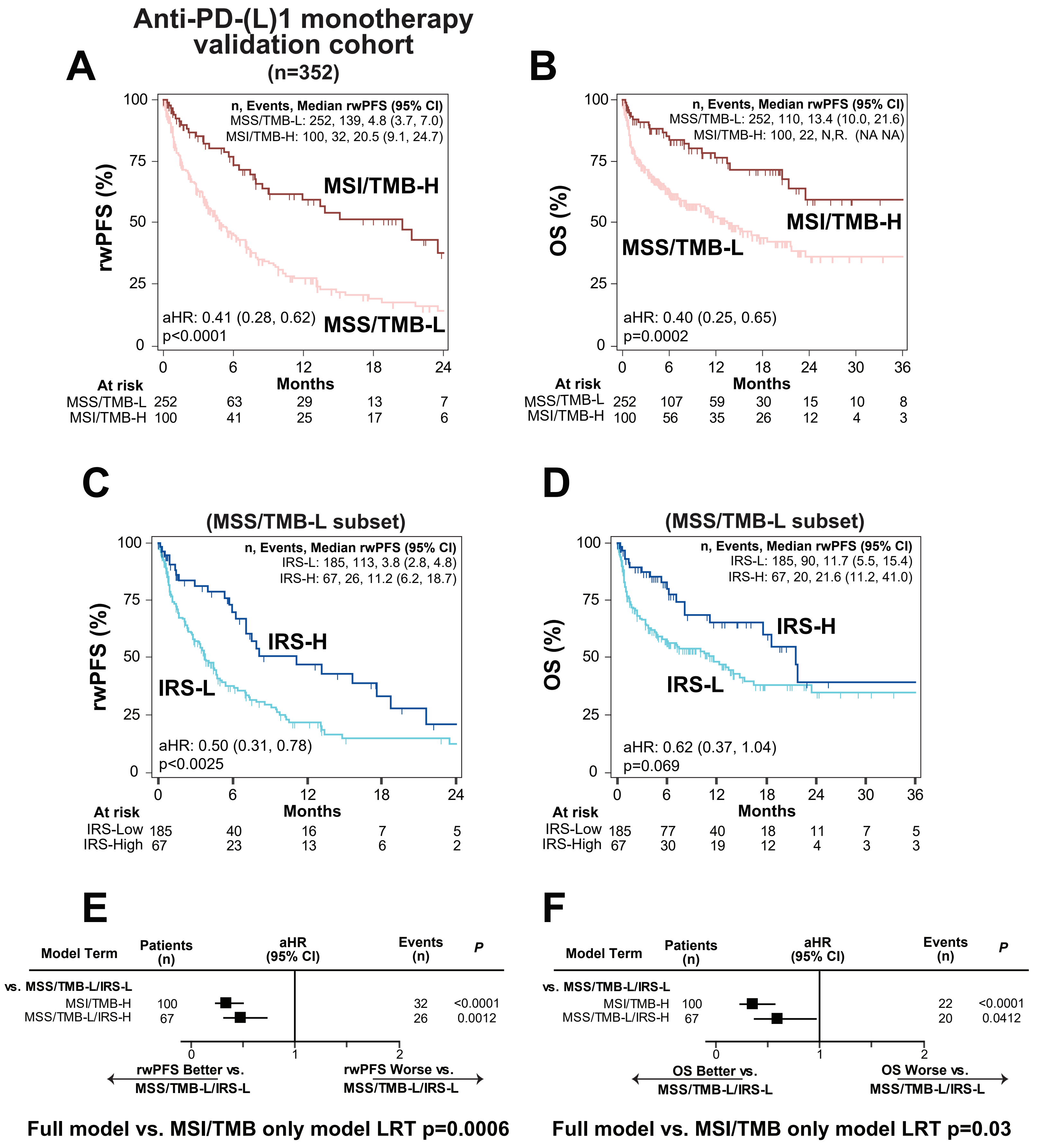

Supplementary Figure S3. Immunotherapy Response Score (IRS) adds to tumor mutation burden (TMB) and microsatellite instability (MSI) status to stratify anti-PD-(L)1 monotherapy clinical benefit

A. The 352 patient anti-PD-(L)1 monotherapy validation cohort (**Figure 1**) was alternatively stratified by MSI and TMB status from clinical comprehensive genomic profiling (MSI-H or TMB-H [MSI/TMB-H] vs. not [MSS/TMB-L]) and anti-PD-(L)1 monotherapy real world progression free survival (rwPFS) is shown by unadjusted Kaplan Meier analysis, and group outcomes compared by Cox proportional hazards (CPH) modeling, with the adjusted hazard ratio (aHR; adjusted as in **Fig 1**), 95% confidence interval (95% CI) and p value for MSI/TMB-H vs. MSS/TMB-L shown. The number (*n*) of patients, events, and median rwPFS (with 95% CI) for each group are shown. **B**. As in **A**, except assessing overall survival (OS). **C**. The MSS/TMB-L group from **A** was further stratified by IRS status (High [-H] vs. Low [-L]) with IRS group outcomes assessed as in **A. D.** As in **C**, except assessing OS. **E**. As in **A**, except using a three-group classification (first MSI-H or TMB-H [MSI/TMB-H], then MSS/TMB-L stratified into MSS/TMB-L/IRS-H and MSS/TMB-L/IRS-L). The forest plots show the aHR for the MSI/TMB-H and MSS/TMB-L/IRS-H terms (MSS/TMB-L/IRS-L as reference) in the CPH model. Significant model terms are shown by filled in aHR estimates. The likelihood ratio test (LRT) p-value comparing the full (including MSI/TMB-H and MSS/TMB-L/IRS-H terms) vs. the reduced (including only the MSI/TMB-H term) model are shown. **F**. As in **E**, except assessing OS.
