# Supplementary material for: Validation of Immunotherapy Response Score as Predictive of Pan-solid Tumor Anti-PD-1/PD-L1 Benefit
Source: Cancer Res Commun. 2023 Jul 25;3(7):1335–49. doi: 10.1158/2767-9764.CRC-23-0036 (PMC10367935; doi:10.1158/2767-9764.CRC-23-0036)
Supplement: Supplementary Figure S4 — shows a validation of the predictive nature of Immunotherapy Response Score [file crc-23-0036-s04.pdf]

Supplementary Figure S4

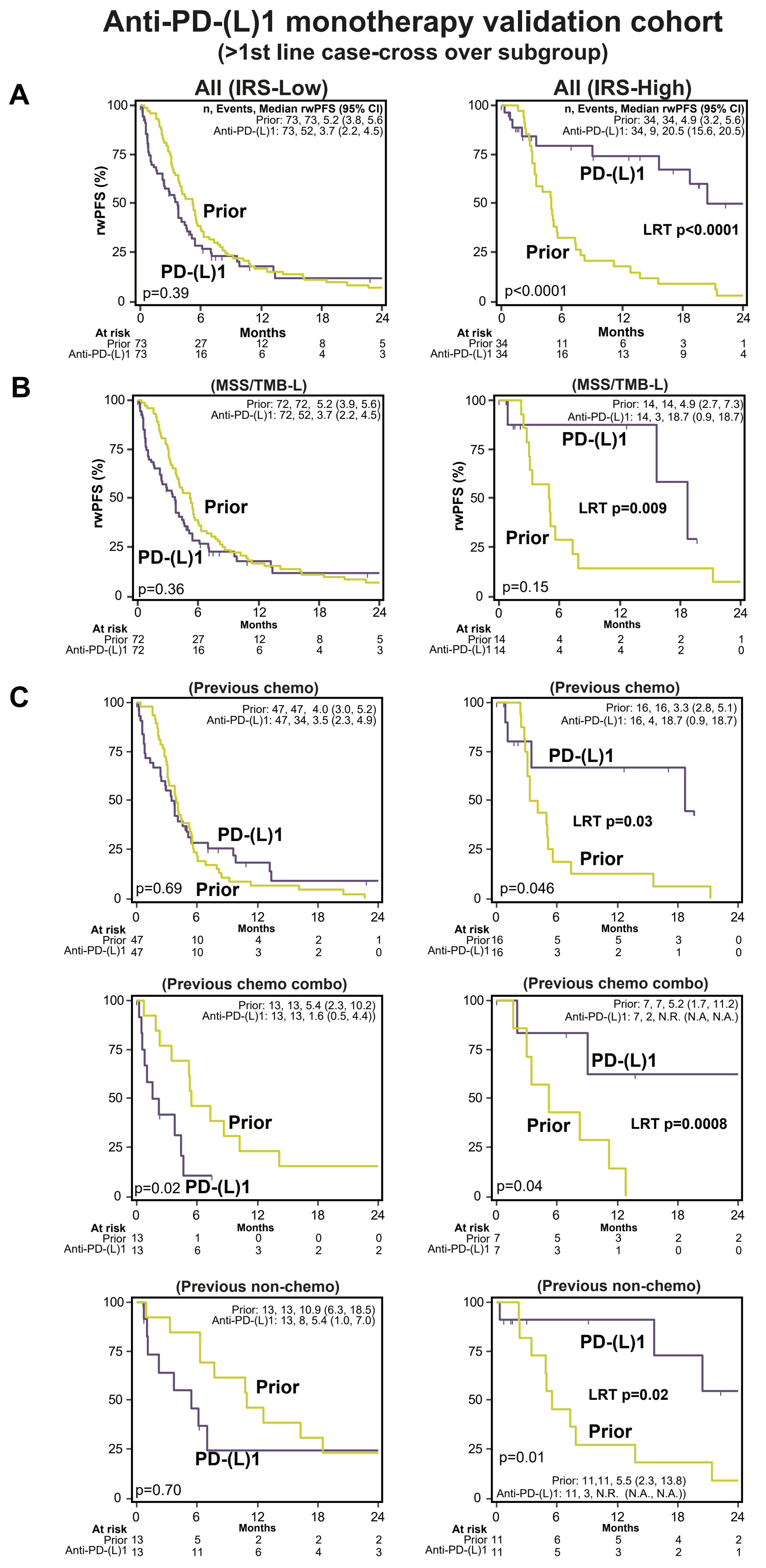

Supplementary Figure S4. Validation of the Predictive Nature of the Immunotherapy Response Score (IRS) Biomarker

To validate the predictive nature of the IRS algorithm, we performed a case cross-over analysis in the n=107 patient subset of the 352 patient anti-PD-(L)1 monotherapy validation cohort (**Fig 1**) who had received a prior line of systemic therapy prior to anti-PD-(L)1 monotherapy (**A**) or the n=86/107 of such patients who were also not microsatellite instable or tumor mutation burden (TMB) high (MSS/TMB-L) by clinical comprehensive genomic profiling (**B**). For each patient, real-world progression free survival (rwPFS) was determined for the line of systemic therapy immediately prior to anti-PD-(L)1 (yellow) and the anti-PD-(L)1 monotherapy line (purple), with rwPFS for each group then stratified by IRS status. **A.** Kaplan-Meier analysis of anti-PD-(L)1 monotherapy rwPFS (purple) vs. prior systemic therapy rwPFS (yellow) in the IRS-Low [L; left] and IRS-High [H; right] subsets of patients (log-rank p-value shown). The number (n) of patients, events, and median rwPFS (with 95% confidence intervals [CI]) for each group are shown. The likelihood ratio test (LRT) p-value for interaction between anti-PD-(L)1vs. immediately prior treatment line and IRS status (-L vs. -H) is also shown. **B**) As in **A**, except limited to the n=86 patients who were MSS/TMB-L. C) As in **A**, except subgroup analysis based on the type of immediately preceding therapy: chemotherapy (chemo) alone (top), chemotherapy combined with another class of therapy (chemo combo; middle), and non-chemotherapy (non-chemo; bottom). N.R. = median rwPFS not reached. N.A. = 95% confidence interval not evaluable.
