# Supplementary material for: Validation of Immunotherapy Response Score as Predictive of Pan-solid Tumor Anti-PD-1/PD-L1 Benefit
Source: Cancer Res Commun. 2023 Jul 25;3(7):1335–49. doi: 10.1158/2767-9764.CRC-23-0036 (PMC10367935; doi:10.1158/2767-9764.CRC-23-0036)
Supplement: Supplementary Figure S5 — shows robustness of Immunotherapy Response Score to self-reported race [file crc-23-0036-s05.pdf]

Supplementary Figure S5

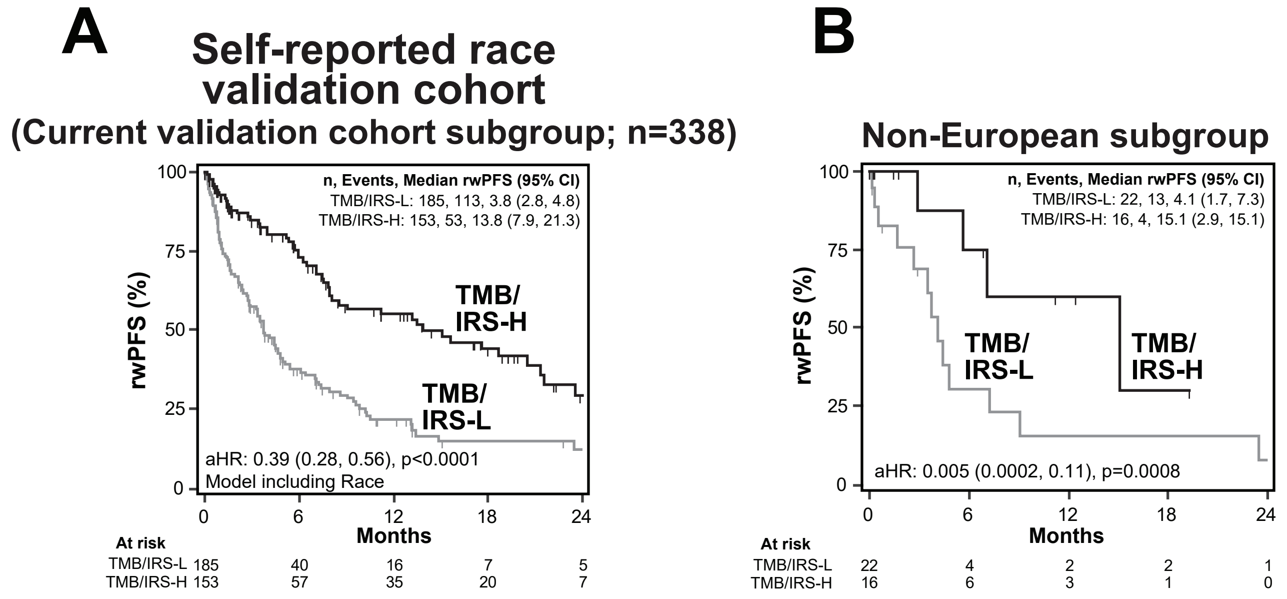

Supplementary Figure S5. Current validation cohort subgroup analysis of the self-reported race validation cohort A. As in **Figure 3D**, except limited to just those eligible patients not included in the previous IRS validation cohort. Anti-PD-(L)1 monotherapy rwPFS stratified by combined TMB and IRS status (TMB-H or IRS-H [TMB/IRS-H; black] vs. TMB-L and IRS-L [TMB/IRS-L; gray] is shown (left) by unadjusted Kaplan Meier analysis, with the adjusted hazard ratio (aHR; adjusted for age, sex assigned at birth, line of therapy, tumor type, anti-PD-[L]1 therapy type, and self-reported race [non-European, Unknown or European), 95% confidence interval (95% CI) and p value for TMB/IRS status (TMB/IRS-H vs. TMB/IRS-L) shown. The number (n) of patients, events, and median rwPFS (with 95% CI) for each group are shown. **B**. Anti-PD-(L)1 monotherapy rwPFS stratified by TMB/IRS status is shown by unadjusted Kaplan Meier analysis for the non-European subgroup of this cohort.
