# Supplementary material for: Validation of Immunotherapy Response Score as Predictive of Pan-solid Tumor Anti-PD-1/PD-L1 Benefit
Source: Cancer Res Commun. 2023 Jul 25;3(7):1335–49. doi: 10.1158/2767-9764.CRC-23-0036 (PMC10367935; doi:10.1158/2767-9764.CRC-23-0036)
Supplement: Supplementary Figure S6 — shows three group Immunotherapy Response Score classification of the anti-PD-(L)1 monotherapy validation cohort [file crc-23-0036-s06.pdf]

Supplementary Figure S6

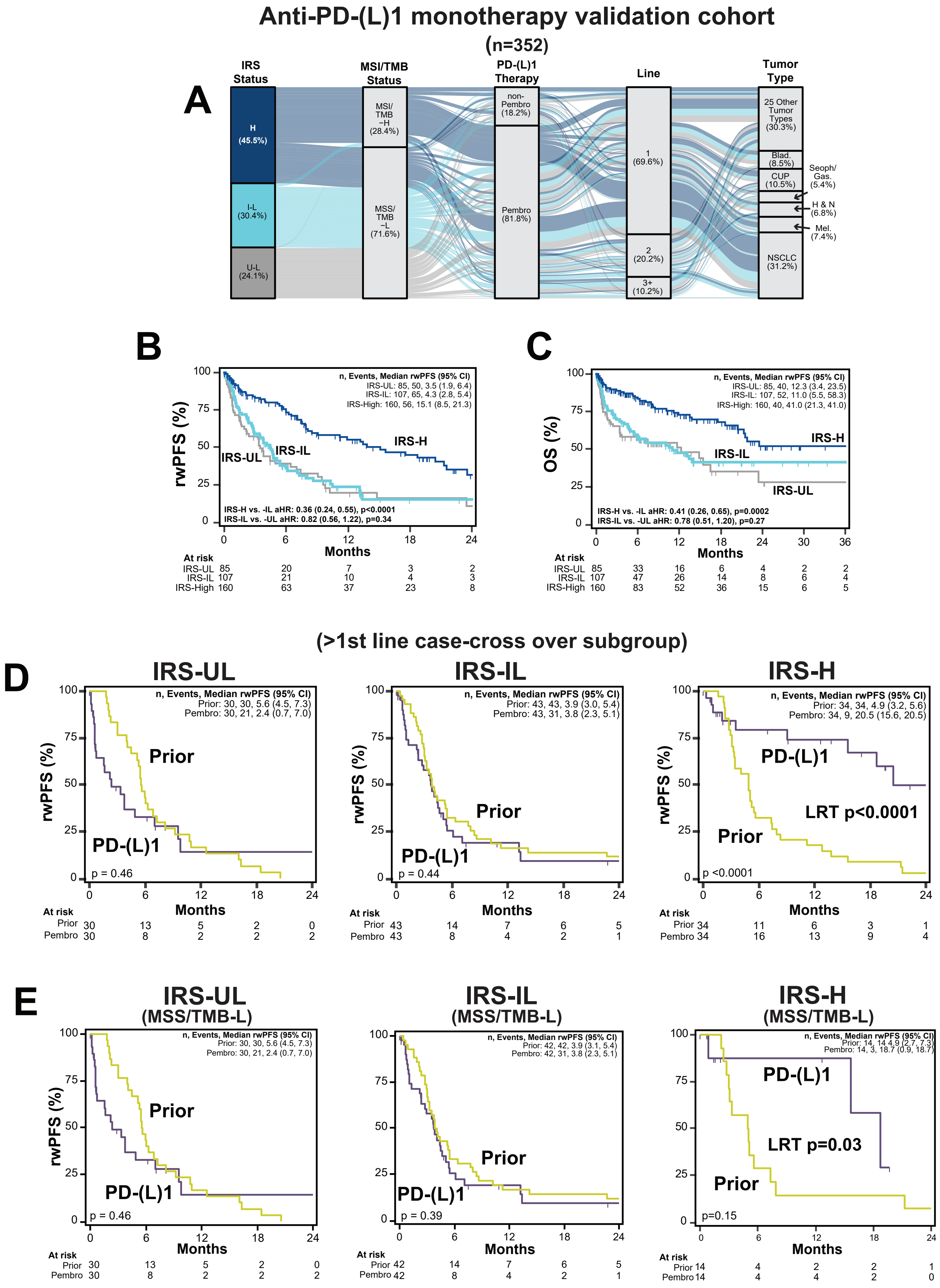

Supplementary Figure S6. Stratification of the anti-PD-(L)1 monotherapy validation cohort by three group Immunotherapy Response Score (IRS) Classification

A. Clinical characteristics of the anti-PD-(L)1 monotherapy validation cohort are shown in an alluvial diagram as in **Figure 1A**, except for the 352 eligible patients, IRS status was assigned by three group IRS classification (IRS-High [-H; dark blue], with IRS-Low divided into IRS-Intermediate Low [-IL; light blue] and IRS-Ultra Low [-UL]). Microsatellite instability (MSI) /TMB status (MSI-H or TMB-H as MSI/TMB-H), type of anti-PD-(L)1 therapy (pembrolizumab [pembro] vs. other anti-PD-[L]1), systemic line of anti-PD-(L)1 therapy, and tumor type (all tumor types with >15 samples considered individually: non small cell lung cancer [NSCLC], cancer of unknown primary [CUP], bladder cancer [Blad.], melanoma [Mel.], head and neck cancer [H&N] and esophagogastric cancer [EGC]; remaining 25 other tumor types considered together) are shown. Stratum are colored by IRS status. **B**. IRS three group classification stratifies anti-PD-(L)1 monotherapy clinical benefit by real-world progression free survival (rwPFS; by time to next therapy). **C**. As in **B**, except overall survival (OS). **D**. Case cross-over analysis as in **Supplementary Figure S4**, except using the three group IRS classification. For each patient, real-world progression free survival (rwPFS) was determined for the line of systemic therapy immediately prior to anti-PD-(L)1 (yellow) and the anti-PD-(L)1 monotherapy line (purple), with rwPFS for each group then stratified by IRS status. Kaplan-Meier analysis of anti-PD-(L)1 monotherapy rwPFS (purple) vs. prior systemic therapy rwPFS (yellow) in the IRS-UL (left), IRS-IL (middle), and IRS-H (right) subsets of patients (log-rank p-value shown). The number (n) of patients, events, and median rwPFS (with 95% confidence intervals [CI]) for each group are shown. The likelihood ratio test (LRT) p-value for interaction between anti-PD-(L)1vs. immediately prior treatment line and IRS status (-L vs. -H) is also shown. **E**. As in **D**, except for the n=86/107 of such patients who were also not microsatellite instable or tumor mutation burden (TMB) high (MSS/TMB-L) by clinical comprehensive genomic profiling**.**

.
