# Supplementary material for: Validation of Immunotherapy Response Score as Predictive of Pan-solid Tumor Anti-PD-1/PD-L1 Benefit
Source: Cancer Res Commun. 2023 Jul 25;3(7):1335–49. doi: 10.1158/2767-9764.CRC-23-0036 (PMC10367935; doi:10.1158/2767-9764.CRC-23-0036)
Supplement: Supplementary Figure S7 — shows covariate adjusted Kaplan-Meier analysis of the chemotherapy, anti-PD-(L)1, and chemotherapy + anti-PD-(L)1 validation cohort [file crc-23-0036-s07.pdf]

Supplementary Figure S7

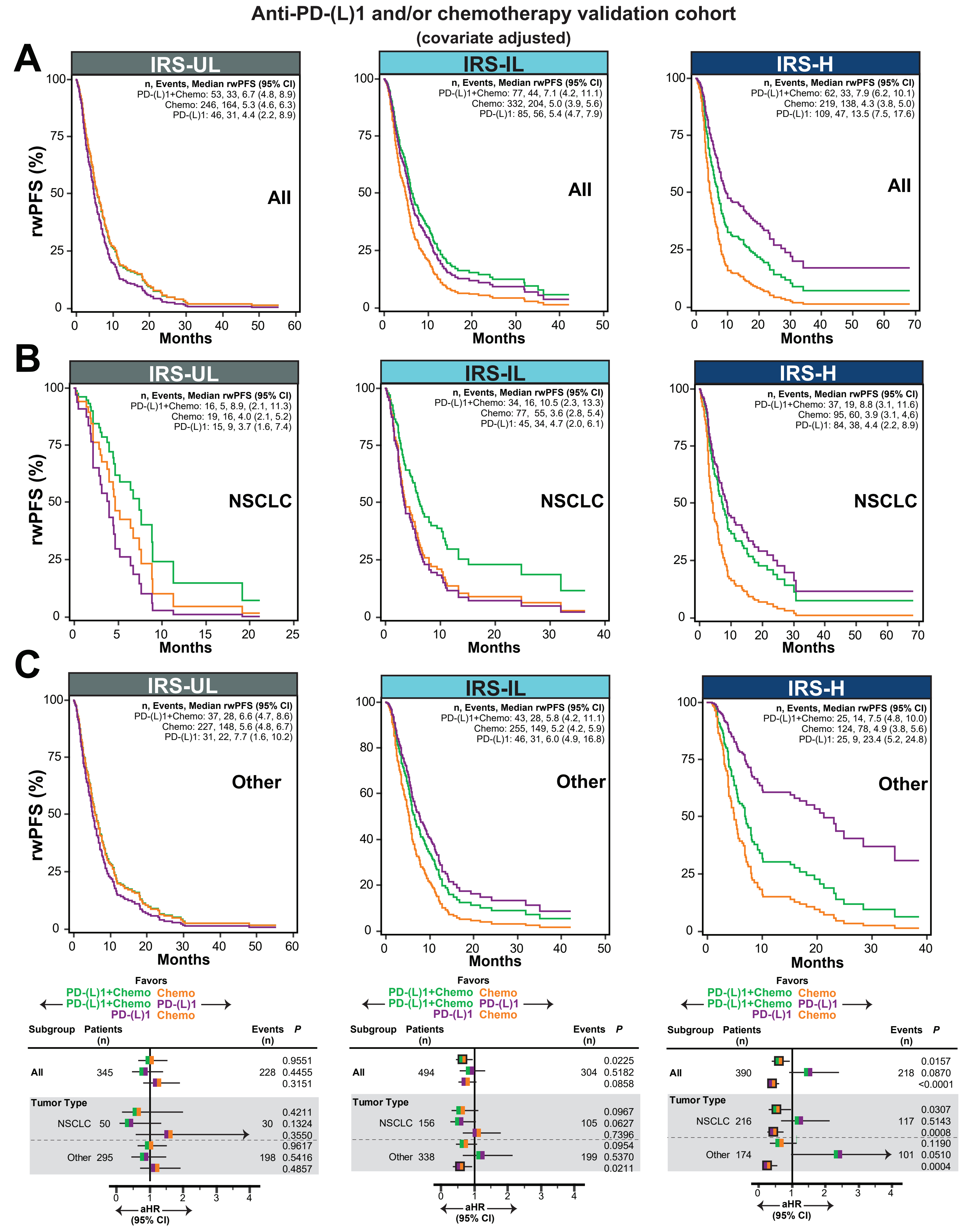

Supplementary Figure S7. Covariate adjusted Kaplan-Meier analysis of the anti-PD-(L)1 and/or chemotherapy (chemo) validation cohort

A. As in **Figure 4B**, except the Kaplan-Meier visualization of the anti-PD-(L)1 and/or chemotherapy (chemo) validation cohort **(**n=1,229 treatment lines) in each Immunotherapy Response Score (IRS) group was performed using covariate adjustment. **B**. As in **A**, except showing the non-small cell lung cancer (NSCLC) subgroup. Forest plot of hazard ratios is shown as in **Figure 4B**. **C**. As in **A**, except showing the other four tumor type (Other; triple negative breast cancer, esophagogastric, head and neck cancer, and small cell lung cancer) subgroups.
