# Supplementary material for: Validation of Immunotherapy Response Score as Predictive of Pan-solid Tumor Anti-PD-1/PD-L1 Benefit
Source: Cancer Res Commun. 2023 Jul 25;3(7):1335–49. doi: 10.1158/2767-9764.CRC-23-0036 (PMC10367935; doi:10.1158/2767-9764.CRC-23-0036)
Supplement: Supplementary Figure S8 — shows overlap weighting propensity score analysis of the chemotherapy, anti-PD-(L)1, and chemotherapy + anti-PD-(L)1 validation cohort [file crc-23-0036-s08.pdf]

Supplementary Figure S8

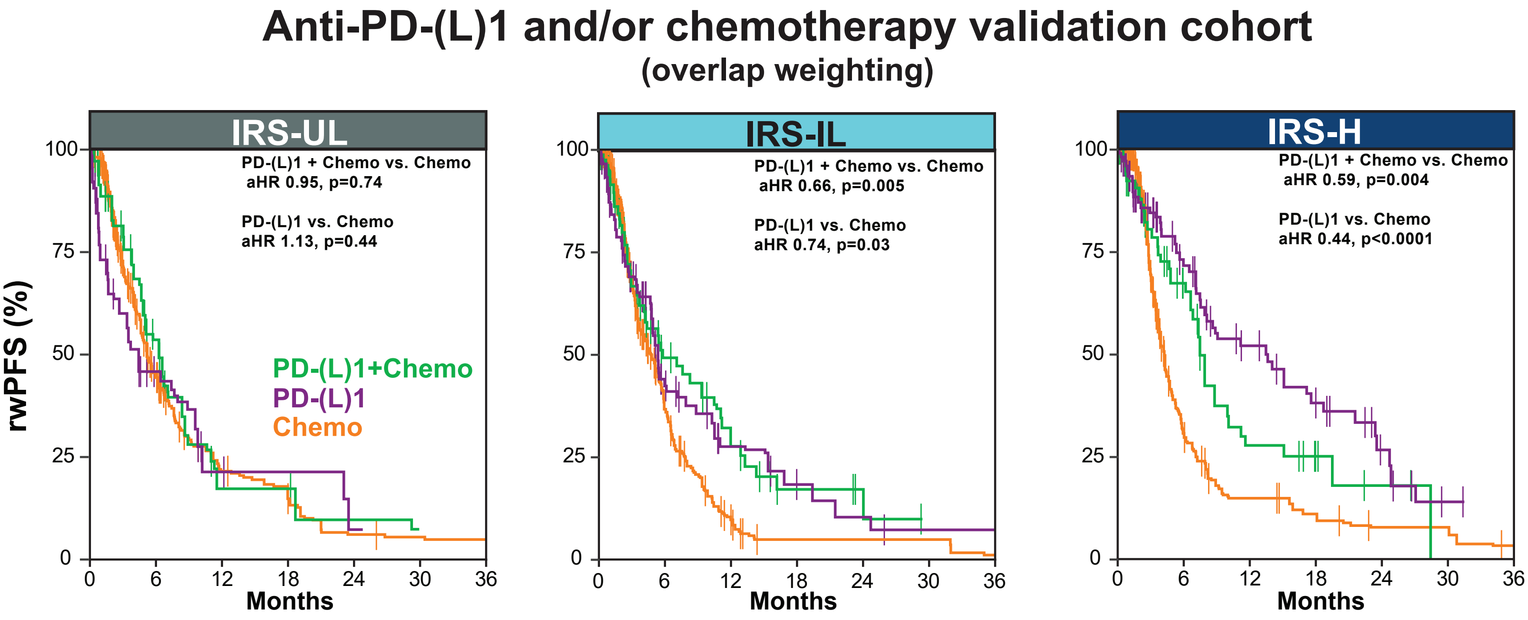

Supplementary Figure S8. Overlap weighting propensity score analysis of the anti-PD-(L)1 and/or chemotherapy (chemo) validation cohort

As in **Figure 4B**, except treatment group rwPFS in each IRS group was compared by overlap weighting-based propensity score analysis using logistic regression to the reference group (chemo alone) on the same variables as used in the adjusted Cox proportional hazards modeling. Propensity scores (PS) were calculated separately within each IRS group; overlap weights (1 - PS for those in the reference group, PS for those outside) were normalized to an average of 1 within each cohort. The adjusted hazard ratio (aHR) and p-value for each comparison to the reference group is shown.
