# Supplementary material for: Validation of Immunotherapy Response Score as Predictive of Pan-solid Tumor Anti-PD-1/PD-L1 Benefit
Source: Cancer Res Commun. 2023 Jul 25;3(7):1335–49. doi: 10.1158/2767-9764.CRC-23-0036 (PMC10367935; doi:10.1158/2767-9764.CRC-23-0036)
Supplement: Supplementary Methods S1 — shows the additional methods and references supporting the main manuscript [file crc-23-0036-s09.pdf]

SUPPLEMENTARY METHODS S1

SUPPLEMENTARY METHODS

Cohort

The Strata Clinical Molecular Database (SCMD) contains deidentified subject, molecular profiling, treatment, and survival data for all enrolled NCT03061305 participants. Prior antineoplastic therapy data, including start and stop dates, were collected for trial participants at the time of study entry. Antineoplastic therapy data and survival status were prospectively collected for up to 3 years from the time of enrollment and/or informed consent. Patient treatment history from electronic health records (EHRs) or manual updating was standardized to enable derivation of real-world progression free survival (rwPFS) by time to next therapy (TTNT) and overall survival (OS) as described(1).

PD-L1 IHC Cohort

We used optical character recognition and natural language processing to prioritize accompanying pathology reports received with StrataNGS test requests for abstraction of IHC biomarker results by trained reviewers according to a documented SOP into a clinical database. For PD-L1, cases were excluded if only categorical results were included, unless reported as <1% (considered 0% for analysis). If a quantitative range of IHC expression was provided, those with range >20% were excluded (e.g., PD-L1 staining reported as >50% were excluded); for results with ≤20% range, the average of the range was used (e.g., PD-L1 TPS >90% = 95%).

PD-L1 expression by the qTP platform is determined through averaging target gene expression from two *PD-L1* amplicon and was performed as described(1). Pearson correlation of qTP *PD-L1* quantification vs. clinical IHC using 276 NSCLC FFPE tumor samples from (1) with clinical PD-L1 IHC expression by the 22C3 clone (using tumor proportion score [TPS]) in accompanying pathology reports was performed after

log2 transformation of the TPS. Pearson correlation of qTP *PD-L1* quantification vs. clinical IHC using 221 FFPE tumor samples (from 23 tumor types with esophagogastric cancer most frequent [35.3%]; only 2.3% NSCLC) with clinical PD-L1 IHC expression by the 22C3 clone (using combined positive score [CPS]) in accompanying pathology reports was performed after $\log_2$ transformation of the CPS. For values reported as CPS or TPS <1%, 0% was used. All correlation analyses used samples with reportable qTP and tumor content ≥20%, but samples were included without respect to treatment information in the SCMD.

To correlate PD-L1 IHC analysis with anti-PD-L1 treatment response, the above TPS and CPS cohorts were limited to just those with valid IRS scores and systemic anti-PD-(L)1 treatments (with or without chemotherapy) who also met the eligibility criteria of the original discovery/validation cohorts (1) or the current validation cohorts (anti-PD-(L)1 monotherapy or anti-PD-(L)1 + chemotherapy), resulting in 177 (CPS n=38, TPS n=139) eligible patients. We also identified 12 eligible patients in the combined discovery and validation cohorts with PD-L1 IHC results by SP142 (reported as immune cell [IC] score) who were included, resulting in a final cohort of 189 patients.

Analytical validity of IRS gene expression components

Analytical validity of IRS as determined by simultaneous comprehensive genomic profiling (CGP; StrataNGS) plus quantitative transcriptional profiling (qTP), including detailed analytical and clinical validation was previously described(1). We subsequently confirmed the accuracy of normalized target gene expression for the four individual IRS RNA expression components *(PD-L1*, *PDCD1*, *TOP2A*, and *ADAM12*) using 96 FFPE intended use tumor specimens (from 24 tumor types) submitted and clinically tested by StrataNGSv4(1) (the qTP expression panel used to generate IRS was run in parallel with clinical StrataNGS testing but not included as a formal analyte).

All samples met the sequencing, input quantity, and input quality criteria defined during the establishment of analytical validity: >150,000 total reads, ≥1ng/ul isolated RNA, tumor surface area (TSA ≥2mm^2) and sample age <5yrs. qRT-PCR was performed from the same RNA aliquot used for clinical StrataNGS testing. For qRT-PCR, 2ul (~9-80ng) clinically isolated FFPE RNA per sample underwent reverse transcription using SuperScript IV VILO Master Mix (Invitrogen) and pre-amplification using TaqMan PreAmp Master Mix (Applied Biosystems) using pooled Taqman primer/hydrolysis probe assays and 10 cycles. qPCR was then performed in duplicate on a Quantstudio 3 Real Time PCR system using a 1:5 dilution of amplified product per qPCR reaction and TaqMan Fast Universal PCR Master Mix (Applied Biosystems). Individual amplicon level thresholds and baselines were set during the exponential amplification phase to determine cycle crossing threshold (C_t [C_q]) values. Samples with duplicate qPCR values > 2 C_t difference and those with both values as undetermined (>40) were excluded. qRT-PCR ΔC_t values were determined as: target amplicon C_t – (*CIAO1* amplicon C_t).

Prior to comparing IRS gene expression component target expression to qRT-PCR, we first plotted IRS target gene expression by qTP vs. the respective comparator amplicon (separate amplicons targeting different exon-exon junctions for *PD-1*, *PD-L1* and *ADAM12*, and an amplicon targeting *UBE2C* [a cell cycle/proliferation gene like *TOP2A*(2-5)] are included for quality control) by qTP to set individual IRS amplicon LOQ lower bounds based on dispersion from linearity; values below LOQ lower bounds were floored to the LOQ lower bound prior to comparison to qRT-PCR ΔC_t values for correlation coefficient and linear fit, as well as formal linearity and LOQ assessment.

We first determined the Pearson correlation coefficient (*r*) and linear fit for normalized target gene expression by quantitative transcriptional profiling vs. normalized target gene expression by qRT-PCR. Minimum Pearson correlation coefficient for each IRS marker was prespecified as ≥0.90. As approaches previously used to validate assay linearity and quantitative bias in multi-gene expression assays not performed in multiplex (e.g., proportionality of threshold cycle [Ct] values vs. input RNA concentration in the validation of Oncotype DX(6,7)) cannot be used directly (following CLSI guidelines) in the

validation of a multiplex RNAseq based-test, we performed an analogous validation.For each gene, linearity was assessed by applying linear (1st order), quadratic (2nd), and cubic (3rd) order polynomial models to normalized target gene expression by StrataNGS+ vs. normalized target gene expression by qRT-PCR. The deviation from linearity (DL) was then calculated from the difference between the linear and best-fitting polynomial prediction (e.g. quadratic order polynomial model) as follows:

$$DLi = p(xi) - (b0+b1xi)$$

where *p(xi)* is the value of the best-fitting polynomial at qRT-PCR determined normalized target gene expression *xi*. As such, *DLi* is a measure of the difference between the nonlinear model and the best-fit straight line at each of the qRT-PCR determined normalized target gene expression values. The upper and lower limits of IRS target gene linearity quantification (LOQ) were selected by establishing the maximum and minimum $\log_2$ nRPM values above and below the median $\log_2$ nRPM value that are > 1.5 DLi (i.e. more than one and a half $\log_2$ unit away from linear prediction). The polynomial models were then re-tested within the selected bounds. Linearity was confirmed if the linear fit was significant ($p<0.05$) but the polynomial terms were not ($p >0.05$). If all four target genes were linear across the entire range of measured values, the within bound Pearson correlation coefficient would be equivalent to that from the entire cohort, and no upper or lower LOQ would be applied for integration into IRS clinical reporting (as done in the discovery and previous validation cohort) as samples were selected to represent the full range of clinical expression observed in >11,000 consecutively tested pan-solid tumor specimens.

Statistical analysis: general considerations

Proportional hazards assumptions were checked for each model and cohort of interest using Schoenfeld residuals. Unstratified analysis results are presented throughout, as stratifying analyses to preserve proportional hazards produced similar covariate effect sizes where the assumption was not met. Unadjusted restricted mean survival time analysis (RMST) was also performed to compare IRS-H and

IRS-L group outcome in the anti-PD-(L)1 monotherapy validation cohort for both rwPFS (at 24 months) and OS (at 36 months), as this analysis does not require the proportional hazards assumption to be met. Performance status (or surrogates) were not available from data collected as part of the Strata Trial.

Statistical analysis: case-control internal comparator cohort predictive analysis

Adjusted Cox proportional hazards models were utilized to examine the interaction between PD-(L)1 vs. prior therapy rwPFS within the same patient and IRS status (IRS-High vs. Low). The likelihood ratio test (LRT) for interaction compared the reduced model (same covariates as the overall cohort except for excluding the anti-PD-(L)1 therapy type and including terms for anti-PD-(L)1 vs. prior therapy), which excluded the IRS by treatment interaction, with the competing full model, which included the IRS by treatment interaction. The two-sided log-rank test was used to test rwPFS differences in the IRS-Low and IRS-High populations. In this cohort, we also determined rwPFS2 (PD-[L]1) / rwPFS1 (immediately prior therapy), and compared the rate of rwPFS2/rwPFS1 ≥1.3 (8-10) in IRS-H vs. IRS-L patients using the Cochran-Mantel-Haenszel test stratified by tumor type (as in the overall monotherapy validation); patients with insufficient PFS2 follow-up (censored prior to 1.3 x PFS1) were excluded. Both analyses were repeated excluding MSI/TMB-H patients. A sub-group analysis based on type of previous therapy (chemotherapy alone, chemotherapy combined with another class, and non-chemotherapy) was also performed for the LRT analysis.

We also determined the interaction between PD-(L)1 vs. prior therapy rwPFS within the same patient and 3 group IRS status (IRS-H, IRS-IL, and IRS-UL); in this analysis, a second dichotomous term was added for IRS-IL, with the likelihood ratio test following a Chi-square distribution with 2 degrees of freedom. This analysis was also performed after excluding MSI/TMB-H patients.

Statistical analysis: Covariate adjustments for the self-reported race validation cohort, PD-L1 IHC cohort, and anti-PD-(L)1 and/or chemo validation cohorts.

For the self-reported race validation cohort, we grouped the included anti-PD-(L)1 monotherapy validation patients into those who were TMB-H or IRS-H [TMB/IRS-H] vs. IRS-L (and TMB-L). Unadjusted rwPFS by IRS/TMB group was visualized using the Kaplan Meier method. Adjusted rwPFS analysis was performed to compare group outcomes (by adjusted hazard ratios and two-sided p-values) exactly as in the overall monotherapy validation cohort, except additionally including a term for inclusion in the previous IRS validation cohort (Yes vs. No) and a self-reported race term (non-European or Unknown [vs. European as the reference]) in the Cox proportional hazards model. Sub-group analysis was also performed in the three self-reported racial groups.

For the PD-(L)1 IHC cohort, adjusted rwPFS analysis was performed to compare outcomes (by adjusted hazard ratios and two-sided p-values) first using a base line model (Model 1) exactly as in the overall monotherapy validation cohort, except excluding the IRS and pembrolizumab vs. other PD-(L)1 therapy terms, and additionally including terms for inclusion in the IRS discovery cohort (Yes vs. No), anti-PD-(L)1 therapy type (monotherapy vs. chemotherapy combination), and PD-L1 IHC (continuous; $\log_2$ reported score [TPS, CPS or IC]). Continuous PD-L1 IHC resulted in a better overall model fit vs. categorical PD-L1 IHC (<1 vs. >=1) and was hence used in the baseline model. To this model, we then added TMB status (-H vs. -L; Model 2) or IRS status (-H vs. -L; Model 3) and compared model performance by the likelihood ratio test comparing the reduced model (Model 1) vs. the full model (Model 2 or 3). We then added IRS status to Model 2 (Model 4) and compared model performance by the likelihood ratio test comparing the reduced model (Model 2) vs. the full model (Model 4). In each model, the aHR of the PD-L1, TMB status, and IRS status terms were determined as appropriate, and unadjusted rwPFS by IRS group was visualized using the Kaplan Meier method. Sensitivity analyses were performed on the full model (Model 4) using the same base Cox model as appropriate for assessment of individual variables (with at least 50 patients per group). Key subgroups included PD-L1 IHC type (TPS vs.

CPS/IC), cohort, anti-PD-(L)1 type (monotherapy vs. chemo combination) and tumor type (NSCLC vs. others).

For the anti-PD-(L)1 and/or chemo validation cohort, unadjusted rwPFS by treatment type was visualized using the Kaplan Meier method separately in each of the 3 category IRS groups (IRS-UL, IRS-IL, and IRS-H). Adjusted rwPFS analysis was performed in each IRS group to compare treatment type outcome (by adjusted hazard ratios and two-sided p-values) controlling for age, gender, line of therapy, tumor type (TNBC as the reference, terms for each of the other four tumor types), and *PD-L1* expression by the qTP platform (categorical tertiles of NSCLC log2 median centered nRPM based expression [<10.3834, 10.3834- 11.6980, >11.6980] in the overall 24,463 SCMD cohort as described ((1)). Both unadjusted and covariate adjusted Kaplan Meier curves were visualized. Sensitivity analyses were performed using the same base Cox model as appropriate for assessment of individual variables (with at least 50 total treatments per IRS group). Overlap weighting-based propensity score analysis (11) was also performed using logistic regression to the reference group (chemotherapy alone) on the same variables as above. Propensity scores were calculated separately within each analysis cohort; overlap weights (1 - PS for those in the reference group, PS for those outside) were normalized to an average of 1 within each cohort.

Statistical analysis: self-reported race analysis

TMB-H (or IRS-H) frequencies in self-reported racial groups in the overall SCMD were compared by two-sided Fisher's exact test (each group vs. White or Caucasian/European). TMB-H (or IRS-H) frequencies per selected tumor type were also compared by two-sided Fisher's exact test between Non-European and European groups. Non-ancestry recalibrated TMB-H frequencies from primary tumor types in the Nassar *et al*. study of the Dana Farber Cancer Institute (DFCI) cohort (provided in **Table S3** of (12)) were calculated for both self-reported racial groups ("Unknown" excluded, "White" considered

European; all other groups considered “Non-European”) and genetically determined ancestry as reported in that study (“European” as reported; Asian or African as “Non-European”).

SUPPLEMENTARY REFERENCES

1. Tomlins SA, Khazanov NA, Bulen BJ, Hovelson DH, Shreve MJ, Lamb LE*, et al.* Development and validation of an integrative pan-solid tumor predictor of PD-1/PD-L1 blockade benefit. *Commun Med (Lond)* 2023;**3**(1):14 doi 10.1038/s43856-023-00243-7.
2. Hovelson DH, Udager AM, McDaniel AS, Grivas P, Palmbos P, Tamura S*, et al.* Targeted DNA and RNA Sequencing of Paired Urothelial and Squamous Bladder Cancers Reveals Discordant Genomic and Transcriptomic Events and Unique Therapeutic Implications. *Eur Urol* 2018;**74**(6):741-53 doi 10.1016/j.eururo.2018.06.047.
3. Plaska SW, Liu CJ, Lim JS, Rege J, Bick NR, Lerario AM*, et al.* Targeted RNAseq of Formalin-Fixed Paraffin-Embedded Tissue to Differentiate Among Benign and Malignant Adrenal Cortical Tumors. *Horm Metab Res* 2020;**52**(8):607-13 doi 10.1055/a-1212-8803.
4. Salami SS, Hovelson DH, Kaplan JB, Mathieu R, Udager AM, Curci NE*, et al.* Transcriptomic heterogeneity in multifocal prostate cancer. *JCI Insight* 2018;**3**(21) doi 10.1172/jci.insight.123468.
5. Tomlins SA, Mehra R, Rhodes DR, Cao X, Wang L, Dhanasekaran SM*, et al.* Integrative molecular concept modeling of prostate cancer progression. *Nat Genet* 2007;**39**(1):41-51 doi 10.1038/ng1935.
6. Cronin M, Sangli C, Liu ML, Pho M, Dutta D, Nguyen A*, et al.* Analytical validation of the Oncotype DX genomic diagnostic test for recurrence prognosis and therapeutic response prediction in node-negative, estrogen receptor-positive breast cancer. *Clin Chem* 2007;**53**(6):1084-91 doi 10.1373/clinchem.2006.076497.
7. Simon R. Roadmap for developing and validating therapeutically relevant genomic classifiers. *J Clin Oncol* 2005;**23**(29):7332-41 doi 10.1200/JCO.2005.02.8712.
8. Palmeri M, Mehnert J, Silk AW, Jabbour SK, Ganesan S, Popli P*, et al.* Real-world application of tumor mutational burden-high (TMB-high) and microsatellite instability (MSI) confirms their utility as immunotherapy biomarkers. *ESMO Open* 2022;**7**(1):100336 doi 10.1016/j.esmoop.2021.100336.
9. Von Hoff DD, Stephenson JJ, Jr., Rosen P, Loesch DM, Borad MJ, Anthony S*, et al.* Pilot study using molecular profiling of patients' tumors to find potential targets and select treatments for their refractory cancers. *J Clin Oncol* 2010;**28**(33):4877-83 doi 10.1200/JCO.2009.26.5983.
10. Massard C, Michiels S, Ferte C, Le Deley MC, Lacroix L, Hollebecque A*, et al.* High-Throughput Genomics and Clinical Outcome in Hard-to-Treat Advanced Cancers: Results of the MOSCATO 01 Trial. *Cancer Discov* 2017;**7**(6):586-95 doi 10.1158/2159-8290.CD-16-1396.
11. Thomas LE, Li F, Pencina MJ. Overlap Weighting: A Propensity Score Method That Mimics Attributes of a Randomized Clinical Trial. *JAMA* 2020;**323**(23):2417-8 doi 10.1001/jama.2020.7819.
12. Nassar AH, Adib E, Abou Alaiwi S, El Zarif T, Groha S, Akl EW*, et al.* Ancestry-driven recalibration of tumor mutational burden and disparate clinical outcomes in response to immune checkpoint inhibitors. *Cancer Cell* 2022;**40**(10):1161-72 e5 doi 10.1016/j.ccell.2022.08.022.
