# Supplementary material for: Validation of Immunotherapy Response Score as Predictive of Pan-solid Tumor Anti-PD-1/PD-L1 Benefit
Source: Cancer Res Commun. 2023 Jul 25;3(7):1335–49. doi: 10.1158/2767-9764.CRC-23-0036 (PMC10367935; doi:10.1158/2767-9764.CRC-23-0036)
Supplement: Supplementary Table S1 — shows the characteristics of the anti-PD-(L)1 monotherapy cohort [file crc-23-0036-s10.pdf]

Supplementary Table S1. Demographics and tumor type of the anti-PD-(L)1 monotherapy cohort

Variable	Statistic	anti-PD-(L)1 mono validation cohort n (%)
**Age (yrs)**	n	352
	median (IQR)	67 (59-74)
**Sex**	n (%)	
Female		158 (44.9%)
Male		194 (55.1%)
**Race**	n (%)	
Asian		3 (0.9%)
Black or African American		27 (7.7%)
Other		11 (3.1%)
Unknown, Missing, or Not Reported		91 (25.9%)
White or Caucasian		220 (62.5%)
**Ethnicity**	n (%)	
Hispanic or Latino		5 (1.4%)
Not Hispanic or Latino		239 (67.9%)
Unknown, Missing, or Not Reported		108 (30.7%)
**Anti-PD-(L)1 line of therapy**	n (%)	
1		245 (69.6%)
2		71 (20.2%)
3+		36 (10.2%)
**Anti-PD-(L)1 therapy type**	n (%)	
pembrolizumab		288 (81.8%)
other anti-PD-(L)1 therapy		64 (18.2%)
**Primary Tumor Type**	n (%)	
Adrenocortical Carcinoma		1 (0.3%)
Ampullary Carcinoma		1 (0.3%)
Appendiceal Cancer		1 (0.3%)
Bladder Cancer		30 (8.5%)
Breast Cancer		9 (2.6%)
Cancer of Unknown Primary		37 (10.5%)
Cervical Cancer		6 (1.7%)
Colorectal Cancer		14 (4.0%)
Endometrial Cancer		6 (1.7%)
Esophagogastric Cancer		19 (5.4%)
Glioma		1 (0.3%)
Head and Neck Cancer		24 (6.8%)
Hepatobiliary Cancer		5 (1.4%)
Lymphoma		1 (0.3%)
Melanoma		26 (7.4%)
Mesothelioma		1 (0.3%)
Non-Small Cell Lung Cancer		110 (31.2%)
Other Cancer		5 (1.4%)
Ovarian Cancer		4 (1.1%)
Pancreatic Cancer		4 (1.1%)
Penile Cancer		1 (0.3%)
Prostate Cancer		2 (0.6%)
Renal Cell Carcinoma		7 (2.0%)
Salivary Gland Cancer		2 (0.6%)
Sarcoma		10 (2.8%)
Skin Cancer, Non-Melanoma		12 (3.4%)
Small Bowel Cancer		1 (0.3%)
Small Cell Lung Cancer		7 (2.0%)
Thymic Tumor		1 (0.3%)
Thyroid Cancer		1 (0.3%)
Vaginal Cancer		3 (0.9%)

Demographics and tumor types for all included patients in the monotherapy validation cohort including age (in years), sex, self reported race, self reported ethnicity, and the anti-PD-(L)1 line of systemic therapy. The StrataNGS CGP assigned primary tumor type for each patient is provided. n= sample size, %= percentage, IQR = interquartile range. .
