# Supplementary material for: Validation of Immunotherapy Response Score as Predictive of Pan-solid Tumor Anti-PD-1/PD-L1 Benefit
Source: Cancer Res Commun. 2023 Jul 25;3(7):1335–49. doi: 10.1158/2767-9764.CRC-23-0036 (PMC10367935; doi:10.1158/2767-9764.CRC-23-0036)
Supplement: Supplementary Table S2 — shows the adjusted Cox proportional hazards models for real-world progression free survival and overall survival in the monotherapy validation cohort [file crc-23-0036-s11.pdf]

Supplementary Table S2: Adjusted Cox proportional hazards model for anti-PD-(L)1 monotherapy real-world progression free survival (rwPFS) and overall survival (OS) in the monotherapy validation cohort (n=352)

Covariate	rwPFS HR (95% CI)	rwPFS p-value	OS HR (95% CI)	OS p-value
Age (yrs; continuous)	0.995 (0.9809 to 1.0093)	0.4913	0.9937 (0.9779 to 1.0099)	0.4451
Sex (Male vs. Female)	0.7307 (0.5308 to 1.0060)	0.0544	0.7522 (0.5239 to 1.0801)	0.1229
Line of Therapy (continuous)	1.1471 (0.9612 to 1.3689)	0.1282	1.1857 (0.9841 to 1.4286)	0.0732
Tumor Type (Bladder vs. Other)	1.314 (0.6808 to 2.5361)	0.4156	1.2523 (0.5596 to 2.8024)	0.5841
Tumor Type (CUP vs. Other)	1.1033 (0.6087 to 2.0000)	0.7459	1.5385 (0.8092 to 2.9252)	0.1888
Tumor Type (Esophagogastric vs. Other)	1.8144 (0.9607 to 3.4267)	0.0663	3.2582 (1.6792 to 6.3220)	0.0005
Tumor Type (Head and Neck vs. Other)	1.3932 (0.7394 to 2.6252)	0.3049	2.0698 (1.0431 to 4.1070)	0.0375
Tumor Type (Melanoma vs. Other)	0.7624 (0.3582 to 1.6227)	0.4815	0.516 (0.1762 to 1.5111)	0.2275
Tumor Type (NSCLC vs. Other)	2.0562 (1.3541 to 3.1223)	0.0007	2.0547 (1.2671 to 3.3317)	0.0035
anti-PD-(L)1 therapy (Pembrolizumab vs. other)	0.8367 (0.5554 to 1.2606)	0.394	0.7593 (0.4758 to 1.2119)	0.2484
IRS status (High vs. Low)	0.4078 (0.2903 to 0.5730)	<0.0001	0.4742 (0.3211 to 0.7003)	0.0002

Associations of covariates with pembrolizumab rwPFS and OS was performed using Cox proportional hazards modeling adjusting for age (years [yrs]; continuous), sex assigned at birth, line of systemic therapy (continuous), Tumor Type (all tumor types with >15 samples were considered separately; remaining tumor types were grouped into a single category [Other]), anti-PD-(L)1 therapy (pembrolizumab vs. other) and Immunotherapy Response Score (IRS) status (-High vs. -Low). The hazard ratio (HR) for each covariate in the full model in the overall 352 patient cohort (see **Fig. 1B&C**) is shown, along with 95% confidence interval (CI), and p-values.
