# Supplementary material for: Validation of Immunotherapy Response Score as Predictive of Pan-solid Tumor Anti-PD-1/PD-L1 Benefit
Source: Cancer Res Commun. 2023 Jul 25;3(7):1335–49. doi: 10.1158/2767-9764.CRC-23-0036 (PMC10367935; doi:10.1158/2767-9764.CRC-23-0036)
Supplement: Supplementary Table S3 — shows unadjusted restricted mean survival time analysis of for real-world PFS and overall survival in the monotherapy validation cohort [file crc-23-0036-s12.pdf]

Supplementary Table S3. Unadjusted restricted mean survival time (RMST) analysis of anti-PD-(L)1 monotherapy real-world PFS (rwPFS) and overall survival (OS) stratified by IRS status in the monotherapy validation cohort

Endpoint	Restricted Time (tau)	IRS Status	RMST Estimate (95% CI)	p-value
	24 months	IRS-L	7.3 (5.9-8.8)	
rwPFS	24 months	IRS-H	14.2 (12.3-16.0)	<0.0001
	36 months	IRS-L	16.7 (14.2-19.1)	
OS	36 months	IRS-H	24.2 (21.5-26.9)	<0.0001

As in **Figure 1B&C** except unadjusted RMST analysis was used to compare anti-PD-(L)1 monotherapy rwPFS (at 24 months) and OS (at 36 months) stratified by IRS status. p-value is for the test of RMST equality between IRS-L and IRS-H.
