# Supplementary material for: Validation of Immunotherapy Response Score as Predictive of Pan-solid Tumor Anti-PD-1/PD-L1 Benefit
Source: Cancer Res Commun. 2023 Jul 25;3(7):1335–49. doi: 10.1158/2767-9764.CRC-23-0036 (PMC10367935; doi:10.1158/2767-9764.CRC-23-0036)
Supplement: Supplementary Table S4 — shows the characteristics of the patients used in the case cross-over analysis [file crc-23-0036-s13.pdf]

Supplementary Table S4. Demographics and tumor type of the subset of patients used in the case cross-over analysis (treated with anti-PD-(L)1 monotherapy in the >1st line)

Variable	Statistic	Anti-PD-(L)1 mono case cross-over cohort n (%)
**Age (yrs)**	n	107
	median (IQR)	63 (54-71.5)
**Sex**	n (%)	
Female		53 (49.5%)
Male		54 (50.5%)
**Race**	n (%)	
Asian		2 (1.9%)
Black or African American		8 (7.5%)
Other		3 (2.8%)
Unknown, Missing, or Not Reported		31 (29.0%)
White or Caucasian		63 (58.9%)
**Ethnicity**	n (%)	
Hispanic or Latino		1 (0.9%)
Not Hispanic or Latino		69 (64.5%)
Unknown, Missing, or Not Reported		37 (34.6%)
**Anti-PD-(L)1 line of therapy**	n (%)	
1		0 (0%)
2		71 (66.4%)
3+		36 (33.6%)
**Anti-PD-(L)1 therapy type**	n (%)	
pembrolizumab		82 (76.6%)
other anti-PD-(L)1 therapy		25 (23.4%)
**Primary Tumor Type**	n (%)	
Adrenocortical Carcinoma		1 (0.9%)
Ampullary Carcinoma		1 (0.9%)
Bladder Cancer		7 (6.5%)
Breast Cancer		5 (4.7%)
Cancer of Unknown Primary		15 (14.0%)
Cervical Cancer		4 (3.7%)
Colorectal Cancer		8 (7.5%)
Endometrial Cancer		4 (3.7%)
Esophagogastric Cancer		12 (11.2%)
Glioma		1 (0.9%)
Head and Neck Cancer		8 (7.5%)
Hepatobiliary Cancer		2 (1.9%)
Lymphoma		1 (0.9%)
Melanoma		2 (1.9%)
Non-Small Cell Lung Cancer		15 (14.0%)
Other Cancer		3 (2.8%)
Ovarian Cancer		2 (1.9%)
Pancreatic Cancer		4 (3.7%)
Prostate Cancer		2 (1.9%)
Renal Cell Carcinoma		2 (1.9%)
Sarcoma		5 (4.7%)
Skin Cancer, Non-Melanoma		1 (0.9%)
Small Cell Lung Cancer		1 (0.9%)
Vaginal Cancer		1 (0.9%)
**Preceding therapy line type**	**n (%)**	
Antibody/Antibody drug conjugate		6 (5.6%)
Antibody+chemotherapy		4 (3.7%)
Angiogenesis antibody		2 (1.9%)
Angiogenesis antibody+chemotherapy		12 (11.2%)
Angiogenesis TKI		2 (1.9%)
Antibody+oncogene		1 (0.9%)
CDK4/6 inhibitor		1 (0.9%)
CDK4/6 inhibitor + chemotherapy		1 (0.9%)
Chemotherapy		63 (58.9%)
Chemotherapy+oncogene TKI		2 (1.9%)
Chemotherapy+PARP inhibitor		1 (0.9%)
Hormonal Therapy		1 (0.9%)
Oncogene TKI		9 (8.4%)
PARP inhibitor		2 (1.9%)

Demographics and tumor types for all included patients in the case cross-over analysis (requiring systemic therapy prior to anti-PD-(L)1 monotherapy treatment) including age (in years), sex, self reported race, self reported ethnicity, and the anti-PD-L1 line of systemic therapy. The StrataNGS CGP assigned primary tumor type for each patient is provided. The type of anti-PD-(L)1 therapy is indicated. The type of therapy for the systemic therapy line immediately preceding the anti-PD-(L)1 monotherapy line is reported. n= sample size, %= percentage, std= standard deviation, IQR = interquartile range.
