# Supplementary material for: Validation of Immunotherapy Response Score as Predictive of Pan-solid Tumor Anti-PD-1/PD-L1 Benefit
Source: Cancer Res Commun. 2023 Jul 25;3(7):1335–49. doi: 10.1158/2767-9764.CRC-23-0036 (PMC10367935; doi:10.1158/2767-9764.CRC-23-0036)
Supplement: Supplementary Table S5 — shows rates of rwPFS2/rwPFS1 in the case cross-over analysis [file crc-23-0036-s14.pdf]

Supplementary Table S5. Rates of rwPFS2 (PD-[L]1)/rwPFS1 (immediately preceding therapy) in the case cross-over analysis of patients treated with anti-PD-(L)1 monotherapy in the >1st line

Group/IRS	rwPFS2/rwPFS1 <1.3		Cochran-Mantel-Haenszel		
	<=1.3	>1.3	Odds Ratio	95% CI	P value
**Overall**					
IRS-H	5	13	6.62	2.2-20.2	0.0005
IRS-L	45	14			
**MSS/TMB-L**					
IRS-H	1	3	4.4	1.1-16.9	0.03
IRS-L	45	14			

All patients with rwPFS2 events at <=1.3 rwPFS2/rwPFS1 and those censored at >1.3 rwPFS2/rwPFS1 from the overall case cross-over cohort (n=107) and the MSS/TMB-L subset (n=86) were evaluated for rate of rwPFS2/rwPFS1 >1.3. Rates were compared by Cochran-Mantel-Haenszel odds ratio test stratified by tumor type.
