# Supplementary material for: Validation of Immunotherapy Response Score as Predictive of Pan-solid Tumor Anti-PD-1/PD-L1 Benefit
Source: Cancer Res Commun. 2023 Jul 25;3(7):1335–49. doi: 10.1158/2767-9764.CRC-23-0036 (PMC10367935; doi:10.1158/2767-9764.CRC-23-0036)
Supplement: Supplementary Table S6 — shows the characteristics of the PD-L1 IHC cohort [file crc-23-0036-s15.pdf]

Supplementary Table S6. Demographics of the 189 patient PD-L1 IHC cohort

Variable	Statistic	Overall n (%)
**Age (yrs)**	n	189
	median (IQR)	67 (60-74)
**Sex**	n (%)	
Female		84 (44.4%)
Male		105 (55.6%)
**Race**	n (%)	
Asian		3 (1.6%)
Black or African American		10 (5.3%)
Other		3 (1.6%)
Unknown, Missing, or Not Reported		50 (26.5)
White or Caucasian		123 (65.1%)
**Ethnicity**	n (%)	
Hispanic or Latino		3 (1.6%)
Not Hispanic or Latino		124 (65.6%)
Unknown, Missing, or Not Reported		62 (32.8%)
**Type of Therapy**	n (%)	
anti-PD-(L)1		102 (54.0%)
Chemo + anti-PD-(L)1		87 (46.0%)
**Line of Therapy**	n (%)	
1		145 (76.7%)
2		35 (18.5%)
3+		9 (4.8%)
**Primary Tumor Type**	n (%)	
Bladder Cancer		13 (6.9%)
Breast Cancer		10 (5.3%)
Cancer of Unknown Primary		11 (5.8%)
Cervical Cancer		2 (1.1%)
Esophagogastric Cancer		17 (9.0%)
Head and Neck Cancer		9 (4.8%)
Melanoma		2 (1.1%)
Non-Small Cell Lung Cancer		121 (64.0%)
Other Cancer		2 (1.1%)
Small Cell Lung Cancer		2 (1.1%)
**Cohort**	n (%)	
Discovery		90 (47.6%)
[illegible]		99 (52.4%)
**PD-L1 IHC Type**	n (%)	
PD-L1 22C3 CPS		38 (20.1%)
PD-L1 SP142 IC		12 (6.3%)
PD-L1 22C3 TPS		139 (73.5%)
**PD-L1 IHC Positive (>=1%)**	n (%)	
**Overall**		157 (83.1%)
PD-L1 22C3 CPS		37 (97.4%)
PD-L1 SP142 IC		12 (100.0%)
PD-L1 22C3 TPS		108 (77.7%)
**PD-L1 IHC Score**	median (IQR)	
**Overall**		3.5 (1.6-6.2)
PD-L1 22C3 CPS		6 (4-25)
PD-L1 SP142 IC		2.5 (1-8.75)
PD-L1 22C3 TPS		20 (2-75)
**TMB Status**	n (%)	
Low		140 (74.1%)
High		49 (25.9%)
**IRS Status**	n (%)	
Low		106 (56.1%)
High		83 (43.9%)

Demographics and tumor types for all 189 patients treated with anti-PD-(L)1 monotherapy or chemotherapy (chemo) + anti-PD-(L)1 with avaialble quantitative clinical PD-L1 IHC reported in accompanying pathology reports, including age (in years), sex, self reported race, self reported ethnicity, type of therapy, the anti-PD-(L)1 +/- chemo line of systemic therapy, tumor type, inclusion in discovery or separate validation cohorts, PD-L1 IHC type (and scoring method), % of overall and IHC type specific PD-L1 positivity (>=1%), overall and IHC type specific IHC score, TMB status (>=10 mutations/megabase as High) and IRS status (High vs. Low). n= sample size, %= percentage, IQR = interquartile range.
