# Supplementary material for: Validation of Immunotherapy Response Score as Predictive of Pan-solid Tumor Anti-PD-1/PD-L1 Benefit
Source: Cancer Res Commun. 2023 Jul 25;3(7):1335–49. doi: 10.1158/2767-9764.CRC-23-0036 (PMC10367935; doi:10.1158/2767-9764.CRC-23-0036)
Supplement: Supplementary Table S7 — shows a sub-group analysis of the PD-L1 IHC cohort [file crc-23-0036-s16.pdf]

Supplementary Table S7. Sub-group analysis of the 189 patient PD-L1 IHC cohort

Variable	Subgroup	Model Term	Treatments (n)	aHR	95%CI Lower	95%CI Upper	Events (n)	*P*
Tumor Type	NSCLC	PD-L1 IHC	121	0.915	0.829	1.011	64	0.081
		TMB status		1.058	0.552	2.030		0.8646
		IRS status		0.571	0.319	1.024		0.0601
	Other	PD-L1 IHC	68	1.039	0.876	1.232	40	0.6629
		TMB status		0.877	0.262	2.933		0.8314
		IRS status		0.564	0.210	1.519		0.2571
PD-L1 IHC type	TPS	PD-L1 IHC	139	0.920	0.838	1.009	75	0.0751
		TMB status		0.996	0.535	1.856		0.99
		IRS status		0.651	0.374	1.133		0.1288
	CPS or IC	PD-L1 IHC	50	1.014	0.781	1.317	29	0.9147
		TMB status		0.850	0.159	4.544		0.8491
		IRS status		0.639	0.140	2.920		0.5638
Cohort	Discovery	PD-L1 IHC	90	0.992	0.852	1.156	48	0.9191
		TMB status		1.737	0.685	4.407		0.245
		IRS status		0.373	0.150	0.931		0.0345
	Validation	PD-L1 IHC	99	0.909	0.805	1.025	56	0.1194
		TMB status		0.795	0.360	1.755		0.5704
		IRS status		0.600	0.302	1.192		0.1449
anti-PD-(L)1 therapy	Mono	PD-L1 IHC	102	1.025	0.892	1.176	55	0.7298
		TMB status		0.867	0.406	1.854		0.7135
		IRS status		0.398	0.206	0.767		0.006
	Chemo combo	PD-L1 IHC	87	0.879	0.773	1.000	49	0.0493
		TMB status		1.251	0.486	3.219		0.6428
		IRS status		0.865	0.362	2.068		0.7441
Gender	Female	PD-L1 IHC	84	0.984	0.864	1.120	48	0.8028
		TMB status		0.906	0.389	2.108		0.8186
		IRS status		0.717	0.367	1.402		0.3311
	Male	PD-L1 IHC	105	0.865	0.748	0.999	56	0.0487
		TMB status		1.104	0.446	2.735		0.8304
		IRS status		0.586	0.241	1.423		0.2374
Age	<65 yrs	PD-L1 IHC	83	0.897	0.784	1.027	44	0.1149
		TMB status		0.483	0.190	1.225		0.1254
		IRS status		1.337	0.603	2.966		0.4748
	>=65 yrs	PD-L1 IHC	106	0.897	0.793	1.015	60	0.0852
		TMB status		1.624	0.723	3.647		0.2406
		IRS status		0.255	0.119	0.545		0.0004

Subgroup analysis of the 189 patients treated with anti-PD-(L)1 monotherapy or chemotherapy (chemo) + anti-PD-(L)1 with avaialble quantitative clinical PD-L1 IHC reported in accompanying pathology reports (from **Figure 2**). For all subgroups (those with >=50 per group are shown), the number of patients in the subgroup, the adjusted hazard ratio (aHR), 95% lower and upper confidence intervals (CI), the number of events in the subgroup, and the p-value (those significant in green) are shown .
