# Supplementary material for: Validation of Immunotherapy Response Score as Predictive of Pan-solid Tumor Anti-PD-1/PD-L1 Benefit
Source: Cancer Res Commun. 2023 Jul 25;3(7):1335–49. doi: 10.1158/2767-9764.CRC-23-0036 (PMC10367935; doi:10.1158/2767-9764.CRC-23-0036)
Supplement: Supplementary Table S8 — shows tumor mutation burden and Immunotherapy Response Score status by self -reported race in the Strata Clinical Molecular Database [file crc-23-0036-s17.pdf]

Supplementary Table S8. TMB-High (-H) and IRS-High status by self reported race in the SCMD

Self Reported Race	Total n	% TMB-H	Fisher's exact test *p*	% IRS-H	Fisher's exact test *p*
Asian	1421	6.4%	*<0.0001*	20.7%	1
Black or African American	1501	9.7%	*0.01*	19.3%	0.86
Other	1339	6.9%	*<0.0001*	18.1%	*0.03*
**Sub-total (Non-European)**	**4261**	**7.7%**	***<0.0001***	**19.4%**	**0.08**
Unknown, Missing, or Not Reported	10420	11.1%	*0.049*	21.6%	0.12
**White or Caucasian**	**9782**	**11.9%**	**--**	**20.7%**	**--**
**Total**	**24463**	**10.8%**		**20.9%**	

The frequency of TMB-High -(H) and IRS-H status across self reported racial groups (Other includes those selecting American Indian or Alaskan Native, or Native Hawaiian or Other Pacific Islander groups) from all 24,463 patients in the SCMD (regardless of treatment status) with valid IRS and TMB status at the time of IRS development. Asian, Black or African American, and Other were also considered together as Non-European. The total number of TMB-H and IRS-H per group, along with the percentage TMB-H and IRS-H, are shown. Fisher's exact test p-values for differences between White or Caucasian vs. each group are shown. Significant values are italicized.
