# Supplementary material for: Validation of Immunotherapy Response Score as Predictive of Pan-solid Tumor Anti-PD-1/PD-L1 Benefit
Source: Cancer Res Commun. 2023 Jul 25;3(7):1335–49. doi: 10.1158/2767-9764.CRC-23-0036 (PMC10367935; doi:10.1158/2767-9764.CRC-23-0036)
Supplement: Supplementary Table S9 — shows tumor mutation burden and Immunotherapy Response Score status by self -reported race in relevant tumor types from the Strata Clinical Molecular Database compared to Nasser et al. [file crc-23-0036-s18.pdf]

Supplementary Table S9. TMB-H and IRS-H status by self reported race in relevant tumor types in the SCMD compared to Nasser *et al*.

Tumor Type	This Study (SCMD)								Nassar et al.									
	Total n		% TMB-H			% IRS-H			Total n		% TMB-H			Total n		% TMB-H		
	Self Non-European	Self European	Self Non-European	Self European	Fisher's exact test *p*	Self Non-European	Self European	Fisher's exact test *p*	Self Non-European	Self European	Self Non-European	Self European	Fisher's exact test *p*	Anc. Non-European	Anc. European	Anc. Non-European	Anc. European	Fisher's exact test *p*
Bladder Cancer	99	316	16.2%	16.8%	1	26.3%	25.6%	0.9	20	587	55.0%	52.3%	1	22	585	63.6%	52.0%	0.39
Colorectal Cancer	602	1227	3.8%	7.3%	*0.004*	8.1%	11.2%	*0.048*	200	1612	39.5%	26.6%	***0.0002***	211	1601	38.4%	26.6%	***0.0006***
Esophagogastric Cancer	203	432	7.9%	3.9%	0.053	23.2%	14.8%	***0.01***	67	873	32.8%	14.3%	***0.003***	78	862	29.5%	14.4%	***0.001***
Head and Neck Cancer	81	247	2.5%	6.9%	0.18	27.2%	21.1%	0.28	23	533	47.8%	20.8%	***0.007***	27	529	37.0%	21.2%	0.06
Melanoma	46	469	17.4%	52.0%	*<0.0001*	30.4%	58.2%	*0.0005*	10	632	40.0%	62.0%	0.19	9	633	33.3%	62.1%	0.09
Non-Small Cell Lung Cancer	719	1426	20.4%	26.5%	*0.002*	41.7%	45.3%	0.12	282	2982	45.0%	38.2%	***0.03***	291	2977	44.0%	38.3%	0.07
Renal Cell Carcinoma	83	188	1.2%	0.0%	0.31	28.9%	21.8%	0.22	12	356	8.3%	10.7%	1	17	351	17.6%	10.3%	0.41
**Total**	1833	4305	11.6%	18.5%	*<0.0001*	26.3%	30.1%	*0.003*	614	7575	41.5%	33.5%	***<0.0001***	655	7538	40.0%	33.6%	***0.001***

The frequency of TMB-H and IRS-H status for selected tumor types (as in Nassar *et al*. 2022) stratified by self reported race (White or Caucasian as European; Black or African American, Asian, American Indian or Alaskan Native, Native Hawaiian or Other Pacific Islander, or Other as Non-European) from all 24,463 patients in the SCMD (regardless of treatment status) with valid IRS and TMB status at the time of IRS development. The total number of European and Non-European patients with each tumor type are shown, along with the percentage TMB-H and IRS-H. Fisher's exact test p-values for differences between European vs. Non-Euorpean TMB-H and IRS-H are shown. Significant values are italicized; values where IRS-H or TMB-H is significantly higher in Non-Europeans vs. Europeans are shown in bold italics. Data from Nassar *et al*. uncorrected tumor only TMB (TMB-H at ≥ 10 mutations/Mb, as in StrataNGS/SCMD) is reported similarly, with separately reported results for self reported race and genetically determined ancestry (Anc.).
