# Supplementary material for: Validation of Immunotherapy Response Score as Predictive of Pan-solid Tumor Anti-PD-1/PD-L1 Benefit
Source: Cancer Res Commun. 2023 Jul 25;3(7):1335–49. doi: 10.1158/2767-9764.CRC-23-0036 (PMC10367935; doi:10.1158/2767-9764.CRC-23-0036)
Supplement: Supplementary Table S10 — shows the characteristics of the anti-PD-(L)1 monotherapy self-reported race validation cohort and the non-European subset [file crc-23-0036-s19.pdf]

Supplementary Table S10. Demographics and tumor type of the 575 patient anti-PD-(L)1 monotherapy self-reported race validation cohort and the non-European subset

Variable		Statistic	Anti-PD-(L)1 cohort n (%)	Non-European Subgroup n (%)
**Age (yrs)**		n	575	47
		median (IQR)	66 (59-75)	64 (50.5-71)
**Sex**		n (%)		
	Female		246 (42.8%)	19 (40.4%)
	Male		329 (57.2%)	28 (59.6%)
**Race**		n (%)		
	Asian		3 (0.5%)	3 (6.4%)
	Black or African American		30 (5.2%)	30 (63.8%)
	Other		14 (2.4%)	14 (29.8%)
	Unknown, Missing, or Not Reported		204 (35.5%)	
	White or Caucasian		369 (64.2%)	
**Ethnicity**		n (%)		
	Hispanic or Latino		7 (1.2%)	4 (8.5%)
	Not Hispanic or Latino		364 (63.3%)	39 (83.0%)
	Unknown, Missing, or Not Reported		204 (35.5%)	4 (8.5%)
**Anti-PD-(L)1 line of therapy**		n (%)		
	1		393 (68.3%)	33 (70.2%)
	2		126 (21.9%)	9 (19.1%)
	3+		56 (9.7%)	5 (10.6%)
**Anti-PD-(L)1 therapy type**		n (%)		
	pembrolizumab		275 (47.8%)	33 (70.2%)
	other anti-PD-(L)1 therapy		300 (52.2%)	14 (29.8%)
**Primary Tumor Type**		n (%)		
	Adrenocortical Carcinoma		1 (0.2%)	1 (2.1%)
	Ampullary Carcinoma		1 (0.2%)	
	Appendiceal Cancer		1 (0.2%)	
	Bladder Cancer		45 (7.8%)	3 (6.4%)
	Breast Cancer		17 (3.0%)	2 (4.3%)
	Cancer of Unknown Primary		53 (9.2%)	4 (8.5%)
	Cervical Cancer		7 (1.2%)	
	Colorectal Cancer		12 (2.1%)	2 (4.3%)
	Endometrial Cancer		4 (0.7%)	
	Esophagogastric Cancer		22 (3.8%)	1 (2.1%)
	Glioma		3 (0.5%)	
	Head and Neck Cancer		38 (6.6%)	7 (14.9%)
	Hepatobiliary Cancer		13 (2.3%)	3 (6.4%)
	Lymphoma		2 (0.3%)	
	Melanoma		87 (15.1%)	1 (2.1%)
	Mesothelioma		2 (0.3%)	1 (2.1%)
	Non-Small Cell Lung Cancer		144 (25.0%)	16 (34.0%)
	Other Cancer		8 (1.4%)	2 (4.3%)
	Ovarian Cancer		4 (0.7%)	
	Pancreatic Cancer		4 (0.7%)	1 (2.1%)
	Penile Cancer		1 (0.2%)	
	Prostate Cancer		2 (0.3%)	
	Renal Cell Carcinoma		38 (6.6%)	1 (2.1%)
	Salivary Gland Cancer		2 (0.3%)	
	Sarcoma		13 (2.3%)	2 (4.3%)
	Skin Cancer, Non-Melanoma		19 (3.3%)	
	Small Cell Lung Cancer		22 (3.8%)	
	Thymic Tumor		1 (0.2%)	
	Thyroid Cancer		1 (0.2%)	
	Vaginal Cancer		4 (0.7%)	

Demographics and tumor types for all included patients including age (in years), sex, self reported race, self reported ethnicity, and the anti-PD-(L)1 line of systemic therapy. The StrataNGS CGP assigned primary tumor type for each patient is provided. n= sample size, %= percentage, IQR = interquartile range.
