# Supplementary material for: Validation of Immunotherapy Response Score as Predictive of Pan-solid Tumor Anti-PD-1/PD-L1 Benefit
Source: Cancer Res Commun. 2023 Jul 25;3(7):1335–49. doi: 10.1158/2767-9764.CRC-23-0036 (PMC10367935; doi:10.1158/2767-9764.CRC-23-0036)
Supplement: Supplementary Table S11 — shows the characteristics of the chemotherapy, anti-PD-(L)1, and chemotherapy + anti-PD-(L)1 validation cohort [file crc-23-0036-s20.pdf]

Supplementary Table S11. Demographics and tumor type of the 1,229 line chemotherapy, anti-PD-(L)1, and chemotherapy + anti-PD-(L)1 validation cohort

Variable	Statistic	Overall n (%)	Chemo n (%)	anti-PD-(L)1 n (%)	Chemo + anti-PD-(L)1 n (%)
**Age (yrs)**	n	1,229	797	240	192
	median (IQR)	64 (56-72)	64 (55-71)	64 (58-73)	64 (57.5-72)
**Sex**	n (%)				
Female		630 (51.3%)	411 (51.6%)	123 (51.2%)	96 (50.0%)
Male		599 (48.7%)	386 (48.4%)	117 (48.7%)	96 (50.0%)
**Race**	n (%)				
Asian		19 (1.5%)	14 (1.8%)	2 (0.8%)	3 (1.6%)
Black or African American		112 (9.1%)	77 (9.7%)	18 (7.5%)	17 (8.9%)
Other		22 (1.8%)	12 (1.5%)	6 (2.5%)	4 (2.1%)
Unknown, Missing, or Not Reported		349 (28.4%)	226 (28.4%)	67 (27.9%)	56 (29.2%)
White or Caucasian		727 (59.2%)	468 (58.7%)	147 (61.2%)	112 (58.3%)
**Ethnicity**	n (%)				
Hispanic or Latino		22 (1.8%)	15 (1.9%)	1 (0.4%)	6 (3.1%)
Not Hispanic or Latino		779 (63.4%)	511 (64.1%)	152 (63.3%)	116 (60.4%)
Unknown, Missing, or Not Reported		428 (34.8%)	271 (34.0%)	87 (36.2%)	70 (36.5%)
**Type of Therapy**	n (%)				
Chemo		797 (64.8%)	797 (100%)	0 (0%)	0 (0%)
anti-PD-(L)1		240 (19.5%)	0 (0%)	240 (100.0%)	0 (0%)
Chemo + anti-PD-(L)1		192 (15.6%)	0 (0%)	0 (0%)	192 (100%)
**IRS Group**	n (%)				
Ultralow (UL)		345 (28.1%)	246 (30.9%)	46 (19.2%)	53 (27.6%)
Intermediate Low (UL)		494 (40.2%)	332 (41.7%)	85 (35.4%)	77 (40.1%)
High (H)		390 (31.7%)	219 (27.5%)	109 (45.4%)	62 (32.3%)
**Line of Therapy**	n (%)				
1		916 (74.5%)	602 (75.5%)	168 (70.0%)	146 (76.0%)
2		214 (17.4%)	129 (16.2%)	55 (22.9%)	30 (15.6%)
3+		99 (8.1%)	66 (8.3%)	17 (7.1%)	16 (8.3%)
**Primary Tumor Type**	n (%)				
Triple Negative Breast Cancer		276 (22.5%)	224 (28.1%)	13 (5.4%)	39 (20.3%)
Esophagogastric Cancer		275 (22.4%)	229 (28.7%)	23 (9.6%)	23 (12.0%)
Head and Neck Cancer		154 (12.5%)	110 (13.8%)	38 (15.8%)	6 (3.1%)
Non-Small Cell Lung Cancer		422 (34.3%)	191 (24.0%)	144 (60.0%)	87 (45.3%)
Small Cell Lung Cancer		102 (8.3%)	43 (5.4%)	22 (9.2%)	37 (19.3%)

Demographics and tumor types for all 1,229 included therapy lines (from 1,013 patients) in the chemotherapy (chemo), anti-PD-(L)1, or chemo + anti-PD-(L)1 validation cohort including age (in years), sex, self reported race, self reported ethnicity, the included line of systemic therapy, the type of therapy and IRS group (3 group classifier). The StrataNGS CGP assigned primary tumor type for each patient is provided. n= sample size, %= percentage, IQR = interquartile range.
