# Supplementary material for: Validation of Immunotherapy Response Score as Predictive of Pan-solid Tumor Anti-PD-1/PD-L1 Benefit
Source: Cancer Res Commun. 2023 Jul 25;3(7):1335–49. doi: 10.1158/2767-9764.CRC-23-0036 (PMC10367935; doi:10.1158/2767-9764.CRC-23-0036)
Supplement: Supplementary Table S12 — shows a sub-group analysis of the chemotherapy, anti-PD-(L)1, and chemotherapy + anti-PD-(L)1 validation cohort [file crc-23-0036-s21.pdf]

**Supplementary Table S12. Subgroup analysis of the 1,229 line chemotherapy, anti-PD-(L)1, and chemotherapy + anti-PD-(L)1 validation cohort**

| | | | IRS-UL | | | | | | IRS-IL | | | | | | IRS-H | | | | | |
|---|---|---|---|---|---|---|---|---|---|---|---|---|---|---|---|---|---|---|---|---|
| Variable | Subgroup | Comparison | Treatments (n) | aHR | 95%CI Lower | 95%CI Upper | Events (n) | *P* | Treatments (n) | aHR | 95%CI Lower | 95%CI Upper | Events (n) | *P* | Treatments (n) | aHR | 95%CI Lower | 95%CI Upper | Events (n) | *P* |
| | | PD-(L)1 + Chemo vs. Chemo | | 1.012 | 0.675 | 1.517 | | 0.9551 | | 0.658 | 0.460 | 0.943 | | 0.0225 | | 0.610 | 0.409 | 0.911 | | 0.0157 |
| | All | PD-(L)1 + Chemo vs. PD-(L)1 | 345 | 0.815 | 0.481 | 1.379 | 228 | 0.4455 | 494 | 0.876 | 0.585 | 1.310 | 304 | 0.5182 | 390 | 1.503 | 0.943 | 2.395 | 218 | 0.087 |
| | | PD-(L)1 vs. Chemo | | 1.242 | 0.814 | 1.895 | | 0.3151 | | 0.752 | 0.543 | 1.041 | | 0.0858 | | 0.406 | 0.280 | 0.589 | | <0.0001 |
| | | PD-(L)1 + Chemo vs. Chemo | | 0.819 | 0.478 | 1.404 | | 0.4681 | | 0.654 | 0.437 | 0.979 | | 0.0392 | | 0.591 | 0.376 | 0.929 | | 0.0226 |
| | 1st Line | PD-(L)1 + Chemo vs. PD-(L)1 | 237 | 0.743 | 0.374 | 1.475 | 151 | 0.3953 | 363 | 0.948 | 0.581 | 1.548 | 221 | 0.8317 | 316 | 1.487 | 0.877 | 2.522 | 173 | 0.141 |
| | | PD-(L)1 vs. Chemo | | 1.103 | 0.634 | 1.919 | | 0.7289 | | 0.690 | 0.458 | 1.040 | | 0.0762 | | 0.397 | 0.262 | 0.602 | | <0.0001 |
| **Line of Therapy** | | PD-(L)1 + Chemo vs. Chemo | | 1.663 | 0.877 | 3.153 | | 0.1195 | | 0.690 | 0.305 | 1.557 | | 0.3711 | | 1.270 | 0.456 | 3.534 | | 0.6472 |
| | >1st line | PD-(L)1 + Chemo vs. PD-(L)1 | 108 | 1.237 | 0.527 | 2.905 | 77 | 0.6254 | 131 | 0.664 | 0.299 | 1.472 | 83 | 0.3131 | 74 | 2.496 | 0.717 | 8.681 | 45 | 0.1505 |
| | | PD-(L)1 vs. Chemo | | 1.344 | 0.689 | 2.623 | | 0.3859 | | 1.039 | 0.558 | 1.934 | | 0.9038 | | 0.509 | 0.149 | 1.738 | | 0.2809 |
| | | PD-(L)1 + Chemo vs. Chemo | | 0.620 | 0.194 | 1.986 | | 0.4211 | | 0.607 | 0.337 | 1.094 | | 0.0967 | | 0.551 | 0.321 | 0.946 | | 0.0307 |
| | NSCLC | PD-(L)1 + Chemo vs. PD-(L)1 | 50 | 0.394 | 0.117 | 1.326 | 30 | 0.1324 | 156 | 0.559 | 0.303 | 1.031 | 105 | 0.0627 | 216 | 1.206 | 0.687 | 2.119 | 117 | 0.5143 |
| | | PD-(L)1 vs. Chemo | | 1.576 | 0.601 | 4.128 | | 0.355 | | 1.086 | 0.667 | 1.768 | | 0.7396 | | 0.457 | 0.289 | 0.721 | | 0.0008 |
| | | PD-(L)1 + Chemo vs. Chemo | | 0.990 | 0.656 | 1.495 | | 0.9617 | | 0.699 | 0.459 | 1.065 | 0 | 0.0954 | | 0.628 | 0.349 | 1.127 | | 0.119 |
| | Remaining 4 | PD-(L)1 + Chemo vs. PD-(L)1 | 295 | 0.837 | 0.472 | 1.484 | 198 | 0.5416 | 338 | 1.199 | 0.674 | 2.132 | 199 | 0.537 | 174 | 2.385 | 0.996 | 5.711 | 101 | 0.051 |
| **Tumor Type** | | PD-(L)1 vs. Chemo | | 1.183 | 0.737 | 1.900 | | 0.4857 | | 0.583 | 0.369 | 0.922 | 0 | 0.0211 | | 0.263 | 0.126 | 0.551 | | 0.0004 |
| | | PD-(L)1 + Chemo vs. Chemo | | 0.938 | 0.219 | 4.023 | | 0.9309 | | 1.867 | 0.763 | 4.566 | | 0.1716 | | | | | | |
| | Esophagago. | PD-(L)1 + Chemo vs. PD-(L)1 | 93 | 0.368 | 0.075 | 1.809 | 67 | 0.2184 | 122 | 4.219 | 1.148 | 15.504 | 78 | 0.0302 | | | | | | |
| | | PD-(L)1 vs. Chemo | | 2.551 | 1.228 | 5.299 | | 0.0121 | | 0.442 | 0.173 | 1.129 | | 0.088 | | | | | | |
| | | PD-(L)1 + Chemo vs. Chemo | | 0.990 | 0.625 | 1.568 | | 0.9659 | | 0.549 | 0.322 | 0.936 | | 0.0275 | | | | | | |
| | Remaining 3 | PD-(L)1 + Chemo vs. PD-(L)1 | 202 | 1.318 | 0.641 | 2.710 | 131 | 0.4526 | 216 | 1.131 | 0.576 | 2.222 | 121 | 0.7206 | | | | | | |
| | | PD-(L)1 vs. Chemo | | 0.751 | 0.381 | 1.480 | | 0.4083 | | 0.485 | 0.273 | 0.862 | | 0.0136 | | | | | | |
| | | PD-(L)1 + Chemo vs. Chemo | | 1.004 | 0.592 | 1.702 | | 0.9891 | | 0.928 | 0.562 | 1.532 | | 0.7689 | | 0.990 | 0.567 | 1.728 | | 0.9705 |
| | <65 yrs | PD-(L)1 + Chemo vs. PD-(L)1 | 211 | 0.992 | 0.490 | 2.009 | 141 | 0.9824 | 252 | 1.176 | 0.661 | 2.093 | 148 | 0.5804 | 184 | 2.189 | 1.123 | 4.266 | 98 | 0.0214 |
| **Age** | | PD-(L)1 vs. Chemo | | 1.012 | 0.575 | 1.780 | | 0.9679 | | 0.788 | 0.492 | 1.262 | | 0.3223 | | 0.452 | 0.263 | 0.778 | | 0.0042 |
| | | PD-(L)1 + Chemo vs. Chemo | | 1.093 | 0.556 | 2.145 | | 0.7971 | | 0.468 | 0.277 | 0.790 | | 0.0045 | | 0.362 | 0.199 | 0.658 | | 0.0009 |
| | >=65 yrs | PD-(L)1 + Chemo vs. PD-(L)1 | 134 | 0.604 | 0.254 | 1.438 | 87 | 0.2545 | 242 | 0.637 | 0.353 | 1.150 | 146 | 0.1344 | 206 | 1.072 | 0.551 | 2.086 | 120 | 0.8377 |
| | | PD-(L)1 vs. Chemo | | 1.809 | 0.886 | 3.693 | | 0.1037 | | 0.735 | 0.462 | 1.169 | | 0.1928 | | 0.338 | 0.198 | 0.576 | | 0.0001 |
| | | PD-(L)1 + Chemo vs. Chemo | | 0.691 | 0.334 | 1.430 | | 0.3189 | | 1.245 | 0.737 | 2.102 | | 0.4126 | | 0.548 | 0.312 | 0.964 | | 0.0368 |
| | Male | PD-(L)1 + Chemo vs. PD-(L)1 | 143 | 1.005 | 0.430 | 2.353 | 93 | 0.9901 | 240 | 1.823 | 0.975 | 3.408 | 155 | 0.0601 | 216 | 1.083 | 0.566 | 2.069 | 121 | 0.8103 |
| **Gender** | | PD-(L)1 vs. Chemo | | 0.687 | 0.353 | 1.337 | | 0.2692 | | 0.683 | 0.407 | 1.146 | | 0.149 | | 0.506 | 0.310 | 0.827 | | 0.0066 |
| | | PD-(L)1 + Chemo vs. Chemo | | 1.113 | 0.672 | 1.846 | | 0.677 | | 0.434 | 0.253 | 0.745 | | 0.0025 | | 0.722 | 0.400 | 1.302 | | 0.2786 |
| | Female | PD-(L)1 + Chemo vs. PD-(L)1 | 202 | 0.582 | 0.287 | 1.178 | 135 | 0.1326 | 254 | 0.505 | 0.278 | 0.914 | 149 | 0.0242 | 174 | 2.408 | 1.198 | 4.843 | 97 | 0.0137 |
| | | PD-(L)1 vs. Chemo | | 1.913 | 1.063 | 3.442 | | 0.0304 | | 0.860 | 0.548 | 1.352 | | 0.5142 | | 0.300 | 0.167 | 0.537 | | 0.0001 |
| | | PD-(L)1 + Chemo vs. Chemo | | 1.152 | 0.720 | 1.844 | | 0.5556 | | 0.783 | 0.491 | 1.247 | | 0.3026 | | 0.928 | 0.448 | 1.922 | | 0.8407 |
| | 1 | PD-(L)1 + Chemo vs. PD-(L)1 | 248 | 0.997 | 0.502 | 1.981 | 165 | 0.9934 | 239 | 0.980 | 0.569 | 1.687 | 161 | 0.9421 | 97 | 2.639 | 0.914 | 7.622 | 58 | 0.0729 |
| | | PD-(L)1 vs. Chemo | | 1.155 | 0.643 | 2.076 | | 0.6293 | | 0.799 | 0.489 | 1.305 | | 0.3694 | | 0.352 | 0.143 | 0.866 | | 0.023 |
| | | PD-(L)1 + Chemo vs. Chemo | | | | | | | | 0.445 | 0.190 | 1.040 | | 0.0616 | | 0.872 | 0.426 | 1.785 | | 0.7081 |
| | 2 | PD-(L)1 + Chemo vs. PD-(L)1 | | | | | | | 137 | 0.461 | 0.177 | 1.197 | 70 | 0.1117 | 123 | 2.165 | 0.875 | 5.356 | 69 | 0.0949 |
| | | PD-(L)1 vs. Chemo | | | | | | | | 0.965 | 0.480 | 1.944 | | 0.9213 | | 0.403 | 0.189 | 0.857 | | 0.0183 |
| **PD-L1 qTP expression (tertile)** | | PD-(L)1 + Chemo vs. Chemo | | | | | | | | 0.380 | 0.133 | 1.088 | | 0.0715 | | 0.279 | 0.126 | 0.618 | | 0.0016 |
| | 3 | PD-(L)1 + Chemo vs. PD-(L)1 | | | | | | | 118 | 0.628 | 0.222 | 1.777 | 73 | 0.3811 | 170 | 0.734 | 0.331 | 1.628 | 91 | 0.4473 |
| | | PD-(L)1 vs. Chemo | | | | | | | | 0.604 | 0.309 | 1.181 | | 0.1406 | | 0.380 | 0.225 | 0.642 | | 0.0003 |
| | | PD-(L)1 + Chemo vs. Chemo | | 0.714 | 0.306 | 1.669 | | 0.4373 | | | | | | | | | | | | |
| | PD-L1 tert 2 or 3 | PD-(L)1 + Chemo vs. PD-(L)1 | 97 | 0.557 | 0.215 | 1.445 | 63 | 0.229 | | | | | | | | | | | | |
| | | PD-(L)1 vs. Chemo | | 1.282 | 0.660 | 2.492 | | 0.4638 | | | | | | | | | | | | |

Subgroup analysis of the 1,229 line chemotherapy, anti-PD-(L)1, and chemotherapy + anti-PD-(L)1 validation cohort (from Figure) 4 is shown. For all subgroups (those with >=50 treatment lines per IRS group are shown), the number of treatment lines in the subgroup, the adjusted hazard ratio (aHR), 95% lower and upper confidence intervals (CI), the number of events in the subgroup, and the p-value (those significant in green) are shown. When <50 treatment lines per group were present, remaining groups were combined and shown (as indicated).
